# The endemic plants of Mozambique: diversity and conservation status

**DOI:** 10.3897/phytokeys.136.39020

**Published:** 2019-12-11

**Authors:** Iain Darbyshire, Jonathan Timberlake, Jo Osborne, Saba Rokni, Hermenegildo Matimele, Clayton Langa, Castigo Datizua, Camila de Sousa, Tereza Alves, Alice Massingue, Jeneen Hadj-Hammou, Sonia Dhanda, Toral Shah, Bart Wursten

**Affiliations:** 1 Royal Botanic Gardens, Kew (RBG Kew), Richmond, Surrey, TW9 3AE, UK Royal Botanic Gardens Richmond United Kingdom; 2 30 Warren Lane, East Dean, East Sussex BN20 0EW, UK Unaffiliated East Dean United Kingdom; 3 Instituto de Investigação Agrária de Moçambique (IIAM), P.O. Box 3658, Mavalane, Maputo, Mozambique Instituto de Investigação Agrária de Moçambique Maputo Mozambique; 4 Durrell Institute of Conservation and Ecology, School of Anthropology and Conservation, Marlowe Building, University of Kent, Canterbury, Kent, CT2 7NR, UK University of Kent Canterbury United Kingdom; 5 Department of Biological Sciences, Eduardo Mondlane University, P.O. Box 257, Maputo, Mozambique Eduardo Mondlane University Maputo Mozambique; 6 Lancaster Environment Centre, Lancaster University, Lancaster, LA1 4YQ, UK Lancaster University Lancaster United Kingdom; 7 Herbarium, Nieuwelaan 38, Meise 1860, Belgium Herbarium Meise Belgium

**Keywords:** centre of endemism, checklist, conservation, flora, herbarium, IUCN Red List, range-restricted

## Abstract

An annotated checklist of the 271 strict-endemic taxa (235 species) and 387 near-endemic taxa (337 species) of vascular plants in Mozambique is provided. Together, these taxa constitute c. 9.3% of the total currently known flora of Mozambique and include five strict-endemic genera (*Baptorhachis*, *Emicocarpus*, *Gyrodoma*, *Icuria* and *Micklethwaitia*) and two near-endemic genera (*Triceratella* and *Oligophyton*). The mean year of first publication of these taxa is 1959, with a marked increase in description noted following the onset of the two major regional floristic programmes, the “Flora of Tropical East Africa” and “Flora Zambesiaca”, and an associated increase in botanical collecting effort. New taxa from Mozambique continue to be described at a significant rate, with 20 novelties described in 2018. Important plant families for endemic and near-endemic taxa include Fabaceae, Rubiaceae and Euphorbiaceae s.s. There is a high congruence between species-rich plant families and endemism with the notable exceptions of the Poaceae, which is the second-most species rich plant family, but outside of the top ten families in terms of endemism, and the Euphorbiaceae, which is the seventh-most species rich plant family, but third in terms of endemism. A wide range of life-forms are represented in the endemic and near-endemic flora, with 49% being herbaceous or having herbaceous forms and 55% being woody or having woody forms. Manica Province is by far the richest locality for near-endemic taxa, highlighting the importance of the cross-border Chimanimani-Nyanga (Manica) Highlands shared with Zimbabwe. A total of 69% of taxa can be assigned to one of four cross-border Centres of Endemism: the Rovuma Centre, the Maputaland Centre sensu lato, and the two mountain blocks, Chimanimani-Nyanga and Mulanje-Namuli-Ribaue. Approximately 50% of taxa have been assessed for their extinction risk and, of these, just over half are globally threatened (57% for strict-endemics), with a further 10% (17% for strict-endemics) currently considered to be Data Deficient, highlighting the urgent need for targeted conservation of Mozambique’s unique flora. This dataset will be a key resource for ongoing efforts to identify “Important Plant Areas – IPAs” in Mozambique, and to promote the conservation and sustainable management of these critical sites and species, thus enabling Mozambique to meet its commitments under the Convention on Biological Diversity (CBD).

## Abstract (pt)

Apresenta-se a lista das plantas vasculares de Moçambique, que compreende 271 taxa endémicos (235 espécies) e 387 taxa quase-endémicos (337 espécies). Estes taxa constituem cerca de 9,3% da flora total actualmente conhecida em Moçambique e incluem cinco géneros estritamente endémicos (*Baptorhachis*, *Emicocarpus*, *Gyrodoma*, *Icuria* e *Micklethwaitia*) e dois géneros quase-endémicos (*Triceratella* e *Oligophyton*). O ano médio das primeiras publicações destes taxa é 1959. Um aumento significativo na descrição de espécies novas foi verificado, relacionado com o início de dois projectos regionais, a “Flora of Tropical East Africa” e a “Flora Zambesiaca”, permitindo um esforço maior de colheitas botânicas. Novos taxa têm vindo a ser descritos a um ritmo significativo, com 20 novas espécies descritas em 2018 para a flora de Moçambique. As famílias Fabaceae, Rubiaceae e Euphorbiaceae, incluem importantes taxa endémicos e quase-endémicos. Existe uma estreita relação entre as famílias de plantas com elevado número de espécies e o grau de endemismo, excepção feita às Poaceae, que embora seja a segunda família mais rica em espécies não se posiciona no grupo das dez principais famílias em termos de endemismo. Por outro lado a família Euphorbiaceae, que é a sétima mais rica em espécies, posiciona-se em terceiro lugar quanto ao número de endemismo. A flora endémica apresenta diferentes formas de vida, sendo 49% das espécies herbáceas e 55% lenhosas. A Província de Manica é o local mais rico em taxa quase-endémicos, realçando assim a importância da área transfronteiriça Chimanimani-Nyanga (Manica) entre Moçambique e Zimbabwe. Refira-se ainda que 69% dos taxa encontra-se num dos quatro centros de endemismo transfronteiriços: o Centro do Rovuma, o Centro de Maputaland sensu lato e nas regiões montanhosas de Chimanimani-Nyanga e Mulanje-Namuli-Ribáuè. Cerca de 50% dos taxa foram avaliados quanto ao risco de extinção, estando mais da metade ameaçados globalmente (57% de endemismos) e 10% (17% de endemismos) foram incluídos na categoria Informação Insuficiente (DD), revelando que a maioria das plantas endémicas de Moçambique necessitam de conservação urgente. Este estudo fornece novos dados indispensáveis à identificação das “Áreas Importantes de Plantas – IPAs” em Moçambique, contribuindo ainda para implementar as estratégias de conservação anteriormente estabelecidas pela Convenção sobre a Diversidade Biológica (CBD).

## Introduction

**Figure 1.**
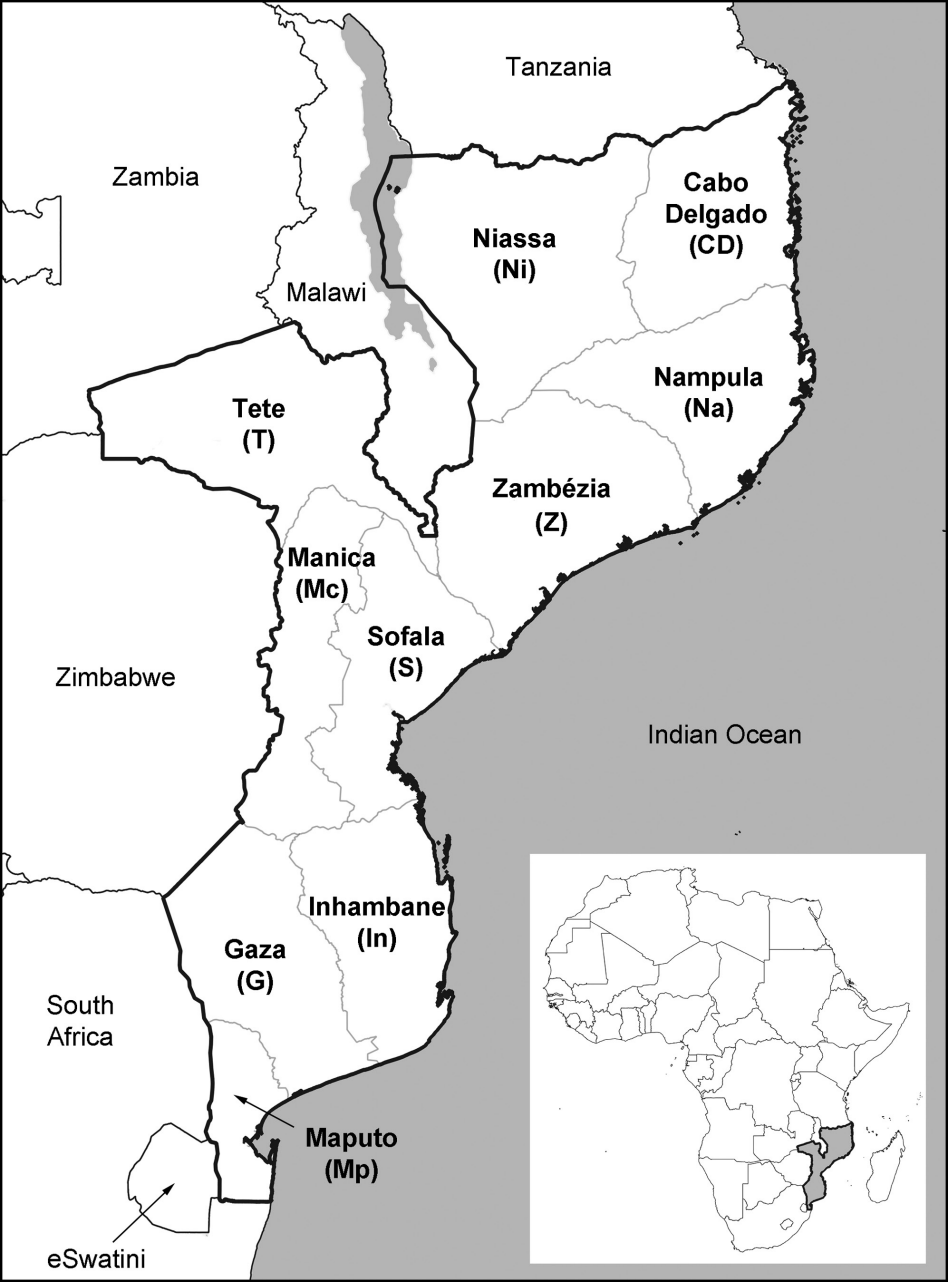
Map of Mozambique showing the ten provinces and neighbouring countries. Provincial borders are shown in pale grey, country borders are in black.

Endemic species are an important component of a country’s biodiversity stewardship and natural capital (Mapaura 2002). Narrowly restricted endemics are often amongst the species most sensitive to environmental change and disturbance, and so at highest risk of extinction (Crisp et al. 2001; Işik 2011; Borokini 2014; Abdelaal et al. 2018; Orsenigo et al. 2018). These species therefore form important components for a range of methods for identifying and conserving biodiversity priorities, such as Important Plant Areas (Darbyshire et al. 2017), Key Biodiversity Areas (IUCN 2016), and the site criteria of the Alliance for Zero Extinction (Ricketts et al. 2005; http://zeroextinction.org/the-alliance/about-the-alliance/). Furthermore, endemic species can be an important consideration when applying the mitigation hierarchy in environmental impact assessments associated with industrial or commercial development projects, particularly at the avoidance and offsetting stages. Therefore, it is important for countries to have an accurate record of their endemic flora, including how many and which species are endemic, and where they are found. At this time of unparalleled rates of biodiversity loss, it is essential to mobilise such information so that countries can effectively prioritise the conservation and sustainable management of their natural resources (Onana 2013). This paper presents the first detailed account of the endemic flora of Mozambique, a biodiversity-rich country in southern tropical Africa (Fig. 1).

### Mozambique: species richness, phytogeography and centres of endemism

Despite its obvious diversity and interest, the flora of Mozambique has received only limited and patchy coverage, particularly when compared to the floras of neighbouring countries. Frodin (2001) estimated the total Mozambican flora as approximately 5,500 species, but noted this was likely to be an under-estimate as “many parts of the country remain imperfectly known” (p. 529). Da Silva et al. (2004) listed only 3,932 indigenous species in their SABONET checklist of Mozambique, of which 177 were noted as endemic. However, it was acknowledged that this list, compiled primarily using specimens held at the LMA and LMU herbaria in Maputo (herbarium acronyms follow Thiers [continuously updated]) with additional records from literature sources, was only provisional, and it has proven to be under-representative. As a good example, da Silva et al. (2004) record nine species of *Barleria* L. (Acanthaceae), but in the “Flora Zambesiaca” (F.Z.) account of Acanthaceae, 33 species of *Barleria* are listed for Mozambique (Darbyshire et al. 2015). In an independent, and more comprehensive analysis, Timberlake et al. (2006) documented 5,692 taxa and 251 endemics in Mozambique including cross-border range-restricted endemics, with an endemism rate of 4.4%. With F.Z. (1960–present) nearing completion, a more accurate measure of species richness in Mozambique is now possible. As of August 2019, the “Flora of Mozambique” website (Hyde et al. 2019a) and associated database of species records, which combine data from F.Z. with updates from relevant literature and field surveys, lists 6,157 native and naturalised species. This figure continues to grow at a rapid rate as targeted botanical surveys of new and botanically interesting areas are conducted, adding new records and new species to science. For example, during surveys of the coastal dry forests in the most north-eastern part of Mozambique in Cabo Delgado Province between 2003 and 2009, during which over 3,000 botanical collections were made, a total of 738 plant taxa were recorded. Of these, 68 were new records for Mozambique, and a further 36 taxa were either entirely new to science or previously known only from fragmentary material and so undescribed (Timberlake et al. 2011).

**Figure 2.**
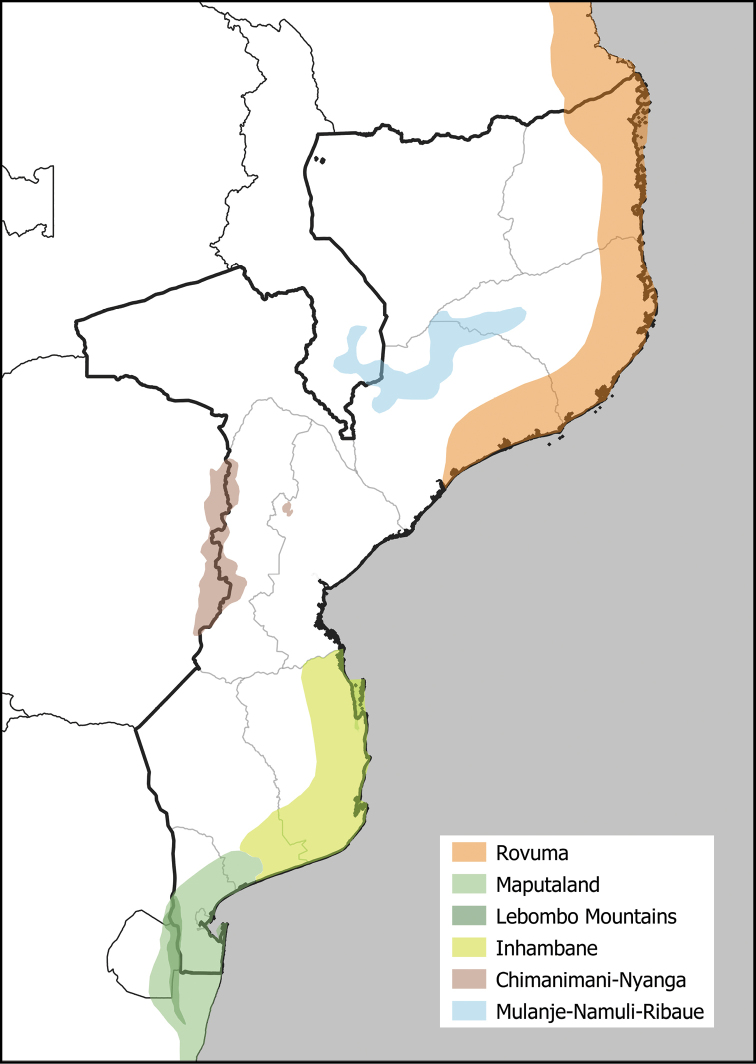
Cross-border Centres of Plant Endemism in Mozambique. Note that the boundaries of these Centres of Endemism are only intended to be indicative; further research is required to more accurately delimit these centres. The two montane Centres (Chimanimani-Nyanga and Mulanje-Namuli-Ribaue) are drawn as continuous blocks for clarity, but in reality they are a discontinuous series of peaks.

Mozambique (Fig. 1) derives its rich and varied plant life in part from its diverse geography, geology and climate, including the influence of its extensive Indian Ocean coastline. These factors have resulted in a wide range of habitats and complex biogeography. Thirteen terrestrial ecoregions are recorded in Mozambique (https://ecoregions2017.appspot.com/; Olson et al. 2001; Burgess et al. 2004; Dinerstein et al. 2017). Moreover, Mozambique features several recognised Centres of Plant Endemism. The majority of the country is included within the Zambezian Regional Centre of Endemism (White 1983), which is widely distributed across southern tropical Africa. Of greater significance in terms of concentrations of range-restricted species, are four cross-border Centres of Endemism (Fig. 2). The first is the recently proposed Rovuma Centre (Burrows and Timberlake 2011) of northeast Mozambique and southeast Tanzania, an extension of the previously recognised Lindi Local Centre in Tanzania (Clarke 2001) or a part of the wider Swahelian Centre of Endemism in coastal East Africa (Clarke 1998). The Rovuma Centre extends along the Mozambique coast through Cabo Delgado, Nampula and Zambézia Provinces approximately as far south as the city of Quelimane (J. Burrows, pers. comm.). The second is the Maputaland Centre (van Wyk 1996; van Wyk and Smith 2001), shared with South Africa and eSwatini (formerly Swaziland), which extends along the coastal lowlands of southern Mozambique to the Limpopo River. This region has several recognised Sub-Centres including the Lebombo Mountains, which straddle the border of the three countries (van Wyk and Smith 2001; Loffler and Loffler 2005). In a wider sense, the Maputaland Centre potentially also extends further northwards from the mouth of the Limpopo River all the way to the mouth of the Save River in Inhambane Province, although this has also been proposed as a putative Centre of Endemism in its own right, the Inhambane Centre (J. Burrows, pers. comm.; A. Massingue, unpubl. data). The third cross-border Centre of Endemism is the Chimanimani-Nyanga (or Manica) Highlands that run along the border with Zimbabwe and form the north-eastern-most extent of the Great Escarpment of southern Africa (Clark et al. 2011). These mountains are well known for their rich floras and high plant endemism (Wild 1964; Mapaura 2002; Clark et al. 2017; Wursten et al. 2017; Cheek et al. 2018). The fourth comprises the larger massifs of the belt of inselbergs running from southern Malawi to Zambézia and Nampula Provinces of northern Mozambique (Bayliss et al. 2014). The most significant peaks are Mount Mulanje (including Mount Mchese) and the Zomba Plateau in Malawi, and Mounts Namuli, Mabu, Inago and the Ribaue Mountains in Mozambique – here shortened to the Mulanje-Namuli-Ribaue Mountains. Mount Mulanje is well established as a site of botanical importance with high endemism (Strugnell 2002, 2006), but the botanical importance of the Mozambique massifs and their links to Mulanje are also becoming increasingly evident (Timberlake et al. 2009, 2012; Harris et al. 2011; Bayliss et al. 2014; Downes and Darbyshire 2017). The latter two Centres form a part of the Africa-wide Afromontane Archipelago-like Centre of Endemism of White (1983).

As these four important Centres of Plant Endemism all cross national borders, it is clearly evident that the political boundary of Mozambique does not reflect species distributions and biogeographic patterns. When considering endemic taxa, therefore, it is pertinent to include within this review those cross-border range-restricted taxa that have a globally significant portion of their range in Mozambique, rather than restricting coverage to taxa that only occur within the political border. Hence the definition of the endemic plants is here extended to include all such relevant near-endemic taxa.

### Motivation for the current study: conservation of the Mozambique flora

In order to address Mozambique’s commitments under the Convention on Biological Diversity (CBD), the “National Strategy and Action Plan of Biological Diversity of Mozambique 2015–2035” (MITADER 2015) sets out a series of detailed national targets for documenting and conserving the biodiversity of Mozambique. Target 6 of this strategy aims to “by 2025, have at least 30% of habitats of endemic and/or threatened flora and fauna species with strategies and action plans for their conservation in place” with a series of related priority actions, including:

• Action 6.1: establish and implement coordinated programs for the systematic assessment of the conservation status of endemic and endangered species;

• Action 6.2: identify and describe the Areas of Plant Importance;

• Action 6.3: disseminate the Red data Book on national flora and fauna.

To address these targets, and to enable effective conservation of Mozambique’s plant diversity in light of increasingly severe pressure on natural resources, a number of botanical initiatives have been launched. A plant Red Listing programme and working group was established in 2011 through the IUCN-SSC Southern African Plant Specialist Group, with the current aim to complete at least 400 new or updated plant species assessments in the period 2017–2020, focussing on strict-endemic and near-endemic species of Mozambique (IUCN SSC Southern African Plant Specialist Group 2017; Matimele 2019). In 2015, the Instituto de Investigação Agrária de Moçambique (the Agrarian Research Institute of Mozambique – IIAM) and the Royal Botanic Gardens, Kew (Kew), together with in-country and international collaborators, launched the “Tropical Important Plant Areas: Mozambique" project (https://www.kew.org/science/projects/tropical-important-plant-areas-tipas-mozambique). This project aims to combine existing data and expertise with targeted field survey data to identify and document Important Plant Areas (IPAs) in Mozambique, and to promote the conservation and sustainable management of these critical sites. This builds on the provisional identification of IPAs through the Southern African Botanical Diversity Network (SABONET) programme (Smith 2005). Further, it draws on the series of extensive botanical surveys in sites of high biodiversity interest across Mozambique that have been conducted by IIAM, Kew and collaborators over the past 15 years. Documentation of the endemic taxa and where they occur is an important step in the IPA and Red Listing programmes, and so provides the motivation for the detailed checklist presented here.

## Materials and methods

### Key resources for compiling the checklist

Compilation of the checklist was based primarily upon extensive reviews of literature on the taxonomy and floristics of Mozambique and neighbouring countries, combined with reference to relevant herbarium collections (notably at BM, BNRH, EA, K, LISC, LMA, LMU, NH, P, PRE and SRGH; herbarium codes follow Thiers [continuously updated]), and the authors’ collective knowledge of the Mozambican flora. A key source for information on the plants of Mozambique, and the starting point for this current work, is the “Flora Zambesiaca” series (F.Z.; 1960–present; http://apps.kew.org/efloras/search.do). This Flora is currently c. 90% complete, with 13 volumes and 47 parts published to date (Exell and Wild 1960, Timberlake and Martins 2015). We have also had access to completed and partially completed accounts for the outstanding volumes: Apocynaceae (Part 2), Commelinaceae, Asteraceae (Compositae) in part, Cyperaceae, and Hyacinthaceae. However, it should be noted that Asteraceae may be under-represented in this checklist in view of the fact that this family has not yet been completed for F.Z.

The “Flora de Moçambique” project ran alongside F.Z. from 1969, but was discontinued in 1981. The accounts in this Flora were derived from F.Z., but with some additional specimen citations and Mozambique-relevant habitat information, thus providing useful additional information for the current work. However, Beentje (2016) estimates that this Flora is less than 40% complete. Other key published works used repeatedly are the recently published landmark volume “Trees and Shrubs [of] Mozambique” (T.S.M.; Burrows et al. 2018); the first national Plant Red List for Mozambique produced through the SABONET programme (S.R.D.L.; Izidine and Bandeira 2002); the field guide to wild flowers of southern Mozambique (Bandeira et al. 1997); and reports on recent botanical surveys and checklists of key localities in Mozambique (Timberlake et al. 2007, 2009, 2010, 2011, 2012, 2016a, 2016b; Bayliss et al. 2010; Harris et al. 2011; Müller et al. 2012; Clark et al. 2017; Wursten et al. 2017). The “Flora of Tropical East Africa” (1952–2012; Beentje 2012, 2016) was also an important source of information for many northern near-endemic species. Key online sources that were widely consulted are the “Flora of Mozambique and Flora of Zimbabwe” sites (Hyde et al. 2019a, 2019b), the African Plant Database (2019), the IUCN Red List of Threatened Species (IUCN 2019), the Botanical Database of Southern Africa / Plants of Southern Africa (South African National Biodiversity Institute 2019), the Red List of South African Plants (South African National Biodiversity Institute 2017) and Plants of the World Online (POWO 2019).

### Definitions of endemism and near-endemism

The taxa treated in the checklist are either strictly endemic to Mozambique (i.e. they only occur within its political borders – labelled E), or are “near-endemic” (NE), as defined by one or more of the following criteria:

(a) the majority of the taxon’s range lies within Mozambique, and they are scarce and/or highly range-restricted beyond (NE1); and/or

(b) the global range of the taxon is less than 10,000 km^2^ (NE2); and/or

(c) the taxon is known globally from five or fewer localities (NE3).

The aim is to include all taxa for which Mozambique has a particularly high responsibility for their global survival and protection, thus those taxa that have the majority of their range in Mozambique, but are also widespread and/or frequent in other parts of southeast tropical Africa are excluded. For example, *Barleria repens* Nees (Acanthaceae) is widely distributed along the East African coast, but with the majority of its distribution in Mozambique because of the vast length of the country’s coastline. However, we do include under (b) and (c) those taxa that do not necessarily have the majority of their range in Mozambique but, because of their highly restricted range and/or scarcity, the Mozambique portion of the population is of global significance to their future survival. We acknowledge that no definition of “near-endemic” is perfect, but we have tried to be as objective as possible when applying the criteria set out above. We have tried to be exhaustive, but our intention is to maintain this list and publish additions and amendments as they are uncovered.

Estimates of range size used in (b) above are based on mapping of known locality data. An offline BRAHMS database (https://herbaria.plants.ox.ac.uk/bol/) of all known collections and sight records of endemic, range-restricted and threatened species is in advanced progress at RBG Kew and IIAM, with approximately 6,000 records compiled to date. Hence, for most of the species on the list we have an accurate measure of range size. For others, where the data are yet to be finalised, ranges have been estimated, aided where available by use of data available via the GeoCAT tool (http://geocat.kew.org/; Bachman et al. 2011); this includes access to relevant GBIF data (GBIF.org 2019). In most cases, the range size is based on the Minimum Convex Polygon (MCP) method commonly applied in the calculation of extent of occurrence (EOO) in the IUCN Red List criteria (Joppa et al. 2016; Bachman et al. 2011; IUCN 2012). However, in a few circumstances where species have highly disjunct distributions with unsuitable habitat in most of the intervening areas, we have estimated range based on the known localities. Of particular note are montane species that are found in the Chimanimani-Nyanga (Manica) Highlands along the Mozambique-Zimbabwe border, but which also extend to Mount Gorongosa, an isolated peak over 100 km to the east in Sofala Province. This usually results in a MCP range of over 10,000 km^2^ (depending on the distribution within the Manica Highlands), but as there is no suitable montane habitat in the intervening region, we treat this range as being less than 10,000 km^2^, and include these species as near-endemics.

### Taxonomy and literature sources

Plant family circumscription follows the Angiosperm Phylogeny Group (APG IV) classification for flowering plants (Stevens 2001 onwards; Angiosperm Phylogeny Group 2016), the Pteridophyte Phylogeny Group (PPG 1; 2016) classification for pteridophytes, and Christenhusz et al. (2011) for gymnosperms. Accepted names of species and infraspecific taxa generally follows the African Plant Database (2019; henceforth APD) except in rare cases where the APD has not been updated to the most recent name, or in the few cases where we disagree with the species circumscription adopted by APD, e.g. *Elaeodendron fruticosum* N.Robson, which is treated as a synonym of *E. matabelicum* Loes. in APD, but we follow Burrows et al. (2018) in recognising it as distinct. Where the taxonomic concept adopted is not universally accepted, or where a taxon has been very recently re-combined, the alternative name is given in brackets. Included on the checklist are all published endemic and near-endemic taxa, together with eight new taxa that are currently either in press or in the late stages of preparation (e.g. *Cyanotis namuliensis* Faden, *Sericanthe chimanimaniensis* Wursten & de Block) such that we are confident of their status. Only species, subspecies and varieties are included in this list; we do not include endemic or near-endemic forms. We have additionally compiled a list of undescribed taxa that are provisionally considered to be endemic or near-endemic to Mozambique, but that have not yet been studied in sufficient detail or are represented by incomplete specimens, for example *Dicliptera* spp. B, C and E of F.Z. (Darbyshire et al. 2015). These are not presented in the checklist, but are available on request from the corresponding author, and included in some of the analyses in the Results and Discussion. Highly doubtful and imperfectly known taxa are excluded. For example, both *Acacia purpurea* Bolle and *Oxyanthus querimbensis* Klotzsch were described from collections made in Mozambique by Wilhelm Peters in the mid-nineteenth century (Peters 1861), and are believed to have been destroyed during the bombing of the Berlin Herbarium in World War II. These species were treated in F.Z. as insufficiently known, and potentially conspecific with other, more widespread species (Brenan 1970; Bridson and Verdcourt 2003).

The date of the original publication (the protologue) is recorded for each taxon. As the aim is to chart the discovery of Mozambique’s endemic flora, it is the date of first publication of the taxon that is of importance, rather than the publication date of the currently accepted name. In many cases these are one and the same, for example *Euphorbia angularis* Klotzsch (in Peters 1861: 92) has been the accepted name ever since its first publication. However, many taxa have changed genus or taxonomic rank since they were first published; for example, the combination for the endemic *Barleria setosa* (Klotzsch) I.Darbysh. was first published in 2015 (Darbyshire et al. 2015), but is based on B. prionitis L. var. setosa Klotzsch, published in Peters (1861: 209), hence 1861 is the date of first publication of this taxon.

For each taxon, we include key references for further information on the plant and its distribution and ecology. Wherever relevant, we include the F.Z. volume and page number, and the page number in T.S.M. and S.R.D.L. For taxa that have been described since the relevant F.Z. volume, we cite the protologue. For those taxa that have changed name or taxonomic rank since F.Z. (for example, have been transferred to a different genus), we cite the relevant F.Z. volume and page number for the taxon account, but also cite the protologue for the currently accepted name.

### Plant life-forms

The growth habit and life cycle of each species are recorded using a simple classification, with six main categories: tree, shrub, liana, herb, pteridophyte and cycad. The herb category is further subdivided into annual (a), perennial (p), succulent-perennial (s), epiphytic-perennial (e), climbing-perennial (c), geophyte (geo), graminoid (gram-a for annual and gram-p for perennial) and seagrass. Trees and shrubs also have a succulent subdivision. Species with variation in growth habit and/or life cycle are recorded in two or more categories.

### Distribution and phytogeography

Taxa known only from the type specimen or type locality are noted. The distribution of each taxon within Mozambique is then recorded, first by scoring which of the provinces it is recorded in (Maputo City Province is included within Maputo Province, hence 10 provinces, Fig. 1), and second by recording key localities in Mozambique arranged by province. The latter are taken from the BRAHMS database noted above, and from additional site observations from the authors. We have attempted to standardise the Mozambican place names, but have used anglicised forms where they are in common use in the literature and/or in gazetteers (such as Mt Mabu and Ribaue Mts, rather than Serra de Mabu and Serra do Ribáuè), and we have avoided use of Portuguese accents on place names, as these are often inconsistently applied. This locality information is provided to help with future study of these species, and to assist with the identification and demarcation of Important Plant Areas. It is not intended to be exhaustive and should not be read as such.

For near-endemic species, the other country (or countries) in which the species occurs is recorded, together with a brief note of key localities; these are not intended to be exhaustive or specific, rather to show how far the species extends beyond Mozambique.

Finally, in order to provide phytogeographic context, the taxa are provisionally assigned where possible to botanical Centres of Endemism (see Introduction). We exclude the widespread Zambezian Regional Centre (White 1983), instead focussing on the more restricted cross-border Centres: (1) Rovuma; (2) Maputaland sensu lato, which we subdivide into (2a) Maputaland sensu stricto (coastal lowlands north to Limpopo River), (2b) Lebombo Mountains (Sub-) Centre, and (2c) Inhambane (Sub-) Centre; (3) Eastern Afromontane, which we subdivide into (3a) Chimanimani-Nyanga (Manica) Highlands, and (3b) Mulanje-Namuli-Ribaue Mountains.

### Extinction risk using the IUCN Red List

Using the categories and criteria of the IUCN Red List (IUCN 2012, 2019), the extinction risk is recorded if the taxon has been assessed; the Red List provides additional information on these species, and so can be considered a further key reference. Red List assessments in need of updating are marked with an asterisk; in most cases these were assessed using an earlier version of the Red List criteria. Red List assessments that have been finalised, but not yet published are listed in italics. Only global Red List assessments are included; we do not list the national assessments of Izidine and Bandeira (2002), as these were highly provisional and are in the process of being re-evaluated on a global scale.

## Results

**Figure 3.**
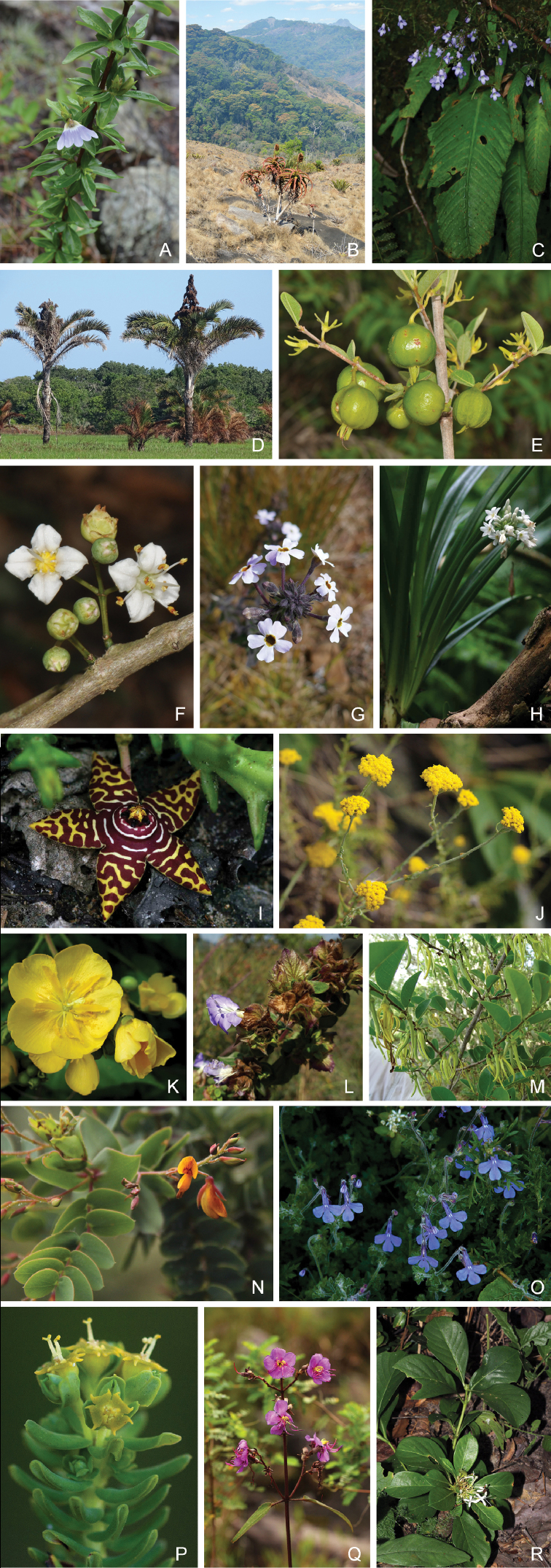
Examples of the strict-endemic and near-endemic plants of Mozambique. **A***Sclerochiton coeruleus*, Maronga, Manica (I. Darbyshire) **B***Aloe ribauensis*, Ribaue, Nampula (I. Darbyshire) **C***Streptocarpus brachynema*, Mount Gorongosa, Sofala (B. Wursten) **D***Raphia australis*, Bilene, Gaza (H. Matimele) **E***Vangueria monteiroi*, Bilene, Gaza (H. Matimele) **F***Memecylon incisilobum*, Bilene, Gaza (H. Matimele) **G***Jamesbrittenia carvalhoi*, Tsetserra, Manica (J. Osborne) **H***Cryptostephanus vansonii*, Mount Gorongosa, Sofala (B. Wursten) **I***Orbea halipedicola*, Gorongosa National Park, Sofala (B. Wursten) **J***Helichrysum moorei*, Chimanimani Mountains, Manica (B. Wursten) **K***Eriolaena rulkensii*, Palma Bay, Cabo Delgado (T. Rulkens) **L***Barleria torrei*, Njesi Plateau, Niassa (J. Osborne) **M***Xylopia torrei*, Licuati Forest, Maputo (H. Matimele) **N***Aeschynomene grandistipulata*, Chimanimani Mountains, Manica (B. Wursten) **O***Lobelia cobaltica*, Chimanimani Mountains, Manica (B. Wursten) **P***Euphorbia crebrifolia*, Chimanimani Mountains, Manica (B. Wursten) **Q***Dissotis pulchra*, Chimanimani Mountains, Manica (B. Wursten) **R***Pavetta pumila*, Cheringoma, Sofala (B. Wursten).

An annotated checklist of the strict-endemic and near-endemic taxa of Mozambique is presented in Suppl. material 1, with a summary of the checklist provided in Appendix 1. It includes all taxa (species, subspecies and varieties) that have been described to date or are in the process of being described. In total, 658 taxa (572 species) are documented, comprising 271 strict-endemic taxa (235 species) and 387 near-endemic taxa (337 species) (Table 1, Fig. 3). In addition, 105 currently undescribed but potentially new taxa (98 species) that are believed to be strict-endemic or near-endemic are noted, but not included in Suppl. material 1 or Appendix 1. If the total number of native and naturalised vascular plant species in Mozambique is taken as ± 6,157 (as per Hyde et al. 2019a), then approximately 3.8% of the species are strict-endemics, whereas the strict-endemics and near-endemics combined account for 9.3% of the plants in Mozambique at the species rank, discounting undescribed taxa. If undescribed taxa are included then approximately 10% of the flora of Mozambique is endemic or near-endemic.

**Table 1.** Summary of endemic taxa in Mozambique. Note that genera are not included in the “Total taxa” row.

**Taxon rank**	**Mozambique strictendemics**	**Mozambique nearendemics**	**Mozambique strict-endemics and near-endemics**
Genus	5	2	7
Species	235	337	572
Subspecies	18	28	46
Variety	18	22	40
**Total taxa**	**271**	**387**	**658**

Mozambique currently has five strict-endemic genera, all of which are monospecific: *Baptorhachis* Clayton & Renvoize (Poaceae) from the granite inselbergs of Nampula Province; *Emicocarpus* K.Schum. & Schltr. (Apocynaceae) from sandy soils around Maputo Bay; *Gyrodoma* Wild (Asteraceae) widespread on alluvial plains, estuaries and margins of lagoons in coastal Mozambique from Zambézia Province southwards; and *Icuria* Wieringa (Fabaceae) and *Micklethwaitia* G.P.Lewis & Schrire (Fabaceae), both occurring as locally dominant trees in the coastal dry forests of northern Mozambique. A further two potential new strict-endemic genera in Asparagaceae (former Hyacinthaceae) are currently under research (T. Rulkens, pers. comm.). In addition, two monospecific genera are near-endemic to Mozambique: *Triceratella* Brenan (Commelinaceae), occurring in moist sands in coastal Zambézia Province, but also known from one locality in Zimbabwe; and *Oligophyton* H.P.Linder & G.Will. (Orchidaceae), restricted to the Chimanimani Mountains on the Zimbabwe-Mozambique border. Two other genera have their sole African representative in Mozambique: *Dolichandrone* Fenzl (Bignoniaceae) and *Eriolaena* DC. (Malvaceae), both of which are predominantly Asian genera (Diniz 1988; Dorr and Wurdack 2018).

Of the near-endemic taxa, 179 are shared with Zimbabwe, 93 with Tanzania, 79 with South Africa, 59 with Malawi, 20 with eSwatini, two with Madagascar and one each with Kenya and Zambia.

**Figure 4.**
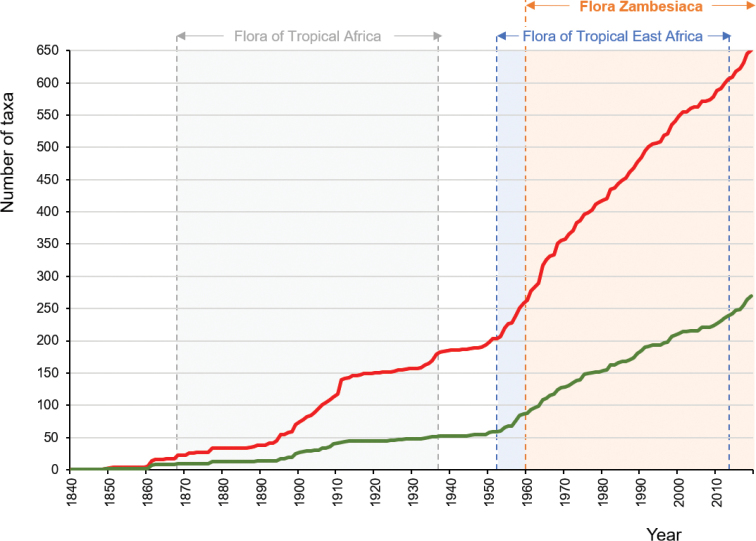
History of publication of the endemic taxa of Mozambique. Cumulative publication dates (basionyms) for currently accepted strict-endemic taxa (green line), and combined strict-endemic and near-endemic taxa (red line), 1840 to present. Also highlighted are the date ranges for the three relevant Tropical African Flora programmes: "Flora of Tropical Africa" (1868–1937), "Flora of Tropical East Africa" (1952–2012) and "Flora Zambesiaca" (1960–present).

Tables 2–6 provide further summaries of the findings presented in Suppl. material 1, namely the most important plant families for strict-endemic and near-endemic taxa (Table 2); the range of life forms of these taxa (Table 3); their geographic distribution by province in Mozambique (Table 4); their distribution within recognised and proposed Centres of Endemism (Table 5); and the extinction risk status of these taxa (Table 6). These tables exclude unpublished taxa. Figure 4 charts the history of publication of the currently accepted strict-endemic and near-endemic taxa in scientific literature.

**Table 2.** Important plant families for published endemic taxa in Mozambique. The 10 plant families with the highest number of endemic taxa, with comparison to the ten most species-rich plant families for the total Mozambican flora (derived from Hyde et al. 2019a). Numbers refer to number of taxa; where two or more plant families share the same number of taxa, the “=” symbol is used to denote that these families have an equal standing in the table.

Mozambique strict-endemics		Mozambique strict-endemics and near-endemics		Total vascular plants of Mozambique	
1. Fabaceae	40	1. Fabaceae	84	1. Fabaceae	759
2. Euphorbiaceae	26	2. Rubiaceae	71	2. Poaceae	445
3. Rubiaceae	23	3. Euphorbiaceae	42	3. Rubiaceae	377
4. Malvaceae	12	4. Lamiaceae	30	4. Asteraceae	352
5. Apocynaceae	11	5.= Apocynaceae	27	5. Orchidaceae	232
6.= Acanthaceae	10	5.= Asteraceae	27	6. Acanthaceae	219
6.= Lamiaceae	10	7. Acanthaceae	26	7.= Euphorbiaceae	194
8. Lythraceae	9	8.= Malvaceae	21	7.= Malvaceae	194
9.= Asphodelaceae	8	8.= Orchidaceae	21	9. Lamiaceae	185
9.= Melastomataceae	8	10. Asphodelaceae	20	10. Apocynaceae	156

**Table 3.** Life forms (growth habits) of published endemic taxa of Mozambique. Note that species can fall under more than one habit category or sub-category. Numbers refer to number of taxa.

Life form (growth habit)		Mozambique strict-endemics	Mozambique strict-endemics and near-endemics
Tree	Non-succulent	54	134
	Succulent	2	9
	**Tree Total**	**56**	**143**
Shrub	Non-succulent	103	283
	Succulent	19	27
	**Shrub Total**	**122**	**310**
Liana		7	28
Woody life forms Total		144	363
Herb	Annual	27	51
	Perennial – non-succulent	67	175
	Perennial -succulent	12	28
	Perennial -epiphyte	1	4
	Perennial – climber/twiner	4	12
	Perennial – geophyte	14	43
	Graminoid – annual	2	4
	Graminoid – perennial	5	11
	Seagrass	1	2
	**Herb Total**	**136**	**324**
Pteridophyte		0	1
Cycad		4	11
Unknown		1	1

**Table 4.** Summary of the geographic distribution of published endemic taxa in the ten provinces of Mozambique. The table is ordered alphabetically by Province; numbers refer to number of taxa.

Province	Mozambique strict-endemics	Mozambique strict-endemics and near-endemics	Provincial endemics	Strict-endemics and near-endemics restricted to one Province
Cabo Delgado (CD)	56	125	27	54
Gaza (G)	26	62	5	7
Inhambane (I)	48	93	15	17
Manica (Mn)	22	192	20	150
Maputo (Mp)	36	119	13	50
Nampula (Na)	86	154	29	38
Niassa (Ni)	19	40	10	21
Sofala (S)	47	105	16	21
Tete (T)	7	18	2	3
Zambézia (Z)	81	159	34	56

**Table 5.** Number of published endemic taxa restricted to Centres and Sub-Centres of Endemism. For the Sub-Centres under (2) Maputaland and (3) [Eastern] Afromontane, taxa are only recorded if they are exclusive to that Sub-Centres.

Centre of Endemism code	(Sub-) Centre of Endemism	Mozambique strict-endemics	Mozambique strict-endemics and near-endemics
1	Rovuma	55	110
2	Maputaland sensu lato (including Inhambane)	50	114
2a	Maputaland sensu stricto	13	32
2b	Lebombo Mountains (Sub-) Centre	3	17
2c	Inhambane (Sub-) Centre	20	20
3	[Eastern] Afromontane sensu lato	46	229
3a	Chimanimani-Nyanga (Sub-) Centre	16	158
3b	Mulanje-Namuli-Ribaue (Sub-) Centre	30	59

**Table 6.** Summary of the extinction risk status of published endemic taxa in Mozambique. The “% of taxa” figure for “Total taxa assessed” is given as a percentage of all the endemic (left) and endemic plus near-endemic (right) taxa listed in Appendix 1; for each of the Red List categories (LC = Least Concern; NT = Near Threatened; VU = Vulnerable; EN = Endangered; CR = Critically Endangered; DD = Data Deficient), the “% of taxa” is given as a percentage of those taxa that have been assessed.

**IUCN Red List Category**	**Mozambique strict-endemics**		**Mozambique strict-endemics and near-endemics**	
	**Number of taxa**	% **of taxa**	**Number of taxa**	% **of taxa**
Total taxa assessed	145	53.5	332	50.5
LC	33	22.8	107	32.2
NT	4	2.8	19	5.7
VU	32	22.1	86	25.9
EN	32	22.1	69	20.8
CR	19	13.1	19	5.7
DD	25	17.2	32	9.6

In Suppl. material 2, we provide a list of taxa that were considered for inclusion in the checklist during its preparation but were ultimately excluded as they did not meet the criteria set out in the Methodology.

For the sake of brevity in the following Discussion, we refer to the combined strict-endemic and near-endemic taxa as “endemics”, whilst we refer to “strict-endemics” if referring only to those taxa unique to Mozambique; the two groups are separated out in the accompanying tables.

## Discussion

### Species richness and endemism in the flora of Mozambique

Based on extrapolation from the RAINBIO mega-database – one of the most comprehensive datasets for plant diversity in tropical Africa, with distribution data for 25,356 native species (Dauby et al. 2016) – Sosef et al. (2017) estimated species richness in Mozambique as between 5,220–5,309 and recorded a relatively high rate of endemism (8.4%, equating to c. 440 spp.) compared to neighbouring countries of southern tropical Africa (Malawi 6.5%, Zambia 7.2%, Zimbabwe 7.6%), although notably lower than Tanzania to the north (19.4%). Current evidence demonstrates that the RAINBIO figure for total species richness is a significant under-estimate, with the total vascular flora currently at 6,157 species (Hyde et al. 2019a), over 15% higher than the upper estimate of Sosef et al. (2017). The known strict-endemism rate of 3.8% is considerably lower than the predicted endemism of Sosef et al. (2017), but if we use the broader definition of endemism applied here to include cross-border near-endemics, then 9.3–10% of taxa are endemics (depending on omission or inclusion of unpublished taxa), which is comparable with the RAINBIO estimate. This figure is considerably higher than the 4.4% endemism rate earlier recorded by Timberlake et al. (2006).

Whilst new discoveries are likely to continue to be made in Mozambique (see below), the percentage endemism of the flora is unlikely to increase, and may even decline as the rate of new country records of non-endemic taxa outstrips the rate of new taxon discovery. For example, in the surveys of the coastal dry forests of northeast Cabo Delgado in 2003–2009, the 68 records of taxa new to Mozambique (Timberlake et al. 2011) included only six near-endemic taxa following the definition applied here. Hence, whilst the discovery of 36 putative new, endemic taxa during these surveys was quite exceptional for eastern tropical Africa in the twenty-first century, it was surpassed at the rate of 1.7:1 by the discovery of new country records of more widespread, non-endemic taxa.

### Discovery of the endemic flora of Mozambique

There have been concerted efforts to document the tropical African flora for over a century and a half, with the first major sub-continental work – the “Flora of Tropical Africa” – dating back to 1868–1937 (Beentje 2016), and the first strict-endemic plant species described in Mozambique as early as 1849 [*Fornasinia ebenifera*Bertolini (1849) = *Millettia ebenifera* (Bertol.) J.E.Burrows & Lötter; see Burrows et al. 2018]. Given these facts, the relatively recent discovery and/or description of many of Mozambique’s endemic plants – the mean year of first publication being 1959, or 1967 for strict-endemics (Fig. 4) – is somewhat surprising. A marked increase in taxon description is observed post-1950, which coincides with the onset of the major eastern African Flora projects – the first fascicle of “Flora of Tropical East Africa” was published in 1952 and the first part of “Flora Zambesiaca” in 1960 (Beentje 2016). Coupled with these Flora projects was major regional-scale botanical exploration to collect herbarium material on which the Flora volumes could be based, and to fill the many gaps in our knowledge of these floristic regions. It was these combined efforts that resulted in the major discoveries of the Mozambique flora, a clear demonstration of how important an active Flora project can be in unlocking information on national and/or regional plant diversity. The completed Floras have, in turn, highlighted localities of high botanical interest, encouraging targeted collecting efforts in Mozambique particularly over the past two decades. Mozambique remains one of the African countries with the highest rates of new species publication. For example, in 2018, 20 new species and one new variety of vascular plants were described from the country, including eight new woody species in the “Trees and Shrubs [of] Mozambique” (Burrows et al. 2018), and four new species of *Memecylon* L. in the Melastomataceae family (Stone et al. 2018).

Of the published endemics, 60 (47 species, 3 subspecies, and 10 varieties) are known only from the type specimen and/or the type locality. This comprises nearly one quarter (22%) of the strict-endemics of Mozambique. A small number of these taxa are of somewhat doubtful status, for example *Teclea crenulata* (Engl.) Engl. (Rutaceae) from Zambézia Province, and some may be subsumed within other, more widespread taxa following further research. However, most are accepted in all relevant taxonomic and floristic works (African Plant Database 2019), and in many cases have been upheld in multiple treatments. The fact that these taxa are so poorly known demonstrates how limited our knowledge of the Mozambique flora remains, and reinforces the likelihood that further discoveries of narrowly range restricted endemics in Mozambique will be made through future botanical exploration.

### Important plant families for endemic and near-endemic taxa in Mozambique

There is generally a high congruence between total species richness per plant family in Mozambique and those families that contain the highest number of endemics, with all but two of the families featuring in both lists of top ten families (Table 2). Fabaceae (Leguminosae) is the most species-rich plant family in Mozambique, and also has the highest number of published endemics. As in most of the African continent, the Fabaceae have diversified significantly in nearly all habitats and ecoregions of Mozambique, and display a large variety of life-forms (Lewis et al. 2005). This, coupled with the high rate of endemism, indicates that the Fabaceae may be considered a suitable proxy group for the study of vascular plant distribution and diversity in Mozambique. Other families that combine high species diversity and high rates of endemism include Acanthaceae, Asteraceae, Malvaceae, Orchidaceae and Rubiaceae. In total, the ten most endemics-rich families contain over half (56%) of the total endemic taxa.

Some species-rich families do not, however, feature prominently in the endemics list, most notably the Poaceae, which is the second largest family in Mozambique, but falls outside the top ten families (twelfth) for endemics. This phenomenon is not isolated to Mozambique, and high proportions of grass taxa globally are known to have large ranges. Linder et al. (2017) noted a range of ecological adaptations that enable grasses to successfully colonise and dominate many ecosystems, including effective long-distance dispersal through wind pollination and seed dispersal, ecological flexibility, resilience to disturbance, and an ability to modify environments by changing fire regimes and mammalian herbivory. Many of these factors could also facilitate wide ranges and abundance of individual grass species.

Conversely, some plant families feature more highly on the endemics list than in terms of total species richness. Euphorbiaceae is the third highest family for endemism, but only equal-seventh for total species richness; this is primarily a result of the high number of range-restricted *Euphorbia* species that occur in Mozambique, most of which are succulents (see Plant life forms below). Furthermore, three plant families feature on the list of families with the highest number of strict-endemics, but not amongst the most species-rich families. The first is Asphodelaceae, which is a result of the high number of *Aloe* L. species. *Aloe* is the single largest genus for endemics in Mozambique with many species being narrowly range-restricted in montane areas and inselbergs (Carter et al. 2011). This is a general trend amongst aloes: while a few species are widespread, the majority have restricted distribution ranges (Reynolds 1950; Grace et al. 2011). The second is Lythraceae, a result of the high number of *Ammannia* L. (including *Nesaea* Comm. ex Kunth.) species that typically occur as small herbs in seasonal wetlands and ephemeral pools. This genus is one of the few groups of aquatic plants to support large numbers of narrowly restricted endemics, with many species known from only one or few collections (see Fernandes 1978; Verdcourt 1994). *Ammannia* should be considered a priority for future study here and elsewhere in tropical Africa with targeted field surveys required in order to better understand the diversity and distribution of this group. The third is Melastomataceae, which is driven largely by the closely related genera *Memecylon* and *Warneckea* Gilg, both of which are primarily forest taxa with high numbers of narrowly range-restricted species throughout their global range (see Stone 2014). As an example, Burrows et al. (2018) note that Namacubi Forest (at Quiterajo in Cabo Delgado Province) is home to seven species in these two genera, three of which are known nowhere else, and a further three of which are strict-endemics or near-endemics to northern Mozambique.

### Plant life forms

A wide range of plant life forms are represented in the checklist (Table 3). Overall, just under half (49%) of taxa listed are herbaceous or have herbaceous forms, whilst just over half (55%) are woody or have woody forms – the small overlap is due to taxa that can be either perennial herbs or shrubs/lianas. Such a range of life forms is unsurprising in view of the wide range of habitats containing endemic and near-endemic species. As with the endemic flora of Zimbabwe (Mapaura 2002), succulent taxa are well represented, with 58 taxa (c. 9%). This reflects the importance of rock outcrops and mountain ranges as key habitats for endemics, as these often support a specialised, drought-tolerant flora.

### Geographic distribution of the endemic and near-endemic taxa of Mozambique

A detailed analysis of the geographic distribution of the endemic flora of Mozambique is premature until the collation of all the specimen and observation data is completed. However, some initial observations can be noted.

By far the most frequently recorded locality for endemics (see Suppl. material 1) is the Chimanimani Mountains (Manica Province, 128 taxa), which has more than double the number of these taxa when compared to the second-most frequently recorded site, Mount Namuli (Zambézia, 60 taxa). The Chimanimani Mountains were also noted as the principal locality in Zimbabwe for strict-endemic and near-endemic species (Mapaura 2002). Other localities rich in endemics, with over 20 taxa each, include Quiterajo, the lower Rovuma River, Quirimbas National Park, and Palma and environs (Cabo Delgado); Pomene and Vilanculos (Inhambane); Tsetserra (Manica); Maputo municipality and Inhaca Island (Maputo); Nampula and environs, and the Ribaue Mountains (Nampula); Gorongosa National Park including Mount Gorongosa (Sofala); and Mocuba and environs (Zambezia). All of these localities are of high national and global importance for their assemblages of endemic and range-restricted taxa, and are clear candidates for inclusion in the Important Plant Areas network, although some have been heavily degraded by man and so are in danger of losing their botanical value. The most notable example is the Maputo municipality, where intact habitats are now reduced to small and isolated pockets, or have been largely destroyed by the rapid expansion of the capital city. Such loss of habitat may have resulted in local extinction of important taxa or, as with *Emicocarpus fissifolius* K.Schum. & Schltr. (Matimele et al. 2016), potentially even global extinction.

There is considerable variation in the number of endemics at the provincial level (Table 4). When only strict-endemics are considered, Nampula and Zambézia provinces register the highest numbers. These two provinces are adjacent to one another and both combine significant stretches of coastal vegetation within the Rovuma Centre of Endemism and inselbergs and massifs associated with the Mulanje-Namuli-Ribaue belt of mountains. The wide range of associated habitats (including coastal dry forest and thickets, granite outcrops, submontane forest, montane grassland) are known to support significant numbers of endemic species. However, when near-endemics are included in the analysis, Manica is found to surpass Nampula and Zambézia in terms of both total numbers of taxa and taxa unique to a single province in Mozambique. This highlights the great importance of the Chimanimani-Nyanga Highlands for cross-border endemism. This also explains the high number of near-endemic taxa shared with Zimbabwe. The least rich province for endemics is Tete, despite being the third largest province in the country. Much of Tete is characterised by a prolonged dry season with extreme high temperatures, and with extensive stands of low-diversity mopane [*Colophospermum mopane* (Benth.) Léonard] woodland. However, it is of note that parts of Tete are amongst the least well-explored regions botanically in Mozambique, and so numbers of endemics may be under-represented in this province.

Approximately 69% of taxa (453) can be assigned with confidence to one of the Centres or Sub-Centres of Endemism (Table 5), highlighting the importance of these mainly cross-border regions in terms of their unique and rich floras. Further, the two sub-centres of the [Eastern] Afromontane phytochorion – the Chimanimani-Nyanga Highlands and the Mulanje-Namuli-Ribaue Mountains – are well-defined, with most species readily assigned to one or the other, strengthening the case for treating them as separate Centres of Endemism. The Lebombo Mountains Sub-Centre of Maputaland is also well-represented by endemics, with 17 of the endemics confined to that Sub-Centre. Similarly, there is support for recognition of the Inhambane Sub-Centre with 20 strict-endemics confined to that region, although there is also considerable overlap between Inhambane and Maputaland sensu stricto, with 42 of the endemics shared between the two regions. Further research may nevertheless conclude that both the Lebombo Mountains and the Inhambane region should be considered as separate Centres of Endemism in their own right. The most important Centre of Endemism for numbers of endemics is again that of the Chimanimani-Nyanga Highlands. However, the Rovuma Centre is also notable for its high number of strict-endemics, a reflection of the high rates of species turnover between dry coastal forest patches within this phytogeographic region (Timberlake et al. 2010, 2011), with many species restricted to few or even single forest blocks.

### Extinction risk in the endemic flora of Mozambique

To date, the global extinction risk status has been assessed for 332 (approximately 50%) of the endemics of Mozambique using the IUCN Red List categories and criteria (IUCN 2012; Table 6). Of those assessed, 52% (57% of the strict-endemics) are considered to be globally threatened. The main causal factors behind this high rate of extinction risk are habitat loss and degradation driven by high population growth and resultant increasing demands for land, agricultural products and supplies of a range of natural resources, all of which place increasing pressure on natural habitats. This high rate of threat emphasises the urgent need for effective site-based conservation action and sustainable management to safeguard the future of Mozambique’s unique flora. Added to this is the fact that nearly 10% of the endemics (and over 17% of the strict-endemics) assessed are listed as Data Deficient, i.e. there is insufficient information on these taxa to provide a full assessment. This highlights how little is known about many of these apparently rare and poorly documented taxa, and the urgent need for targeted field surveys to gather information on range size, population size, and threats. It is quite possible that the percentage of threatened taxa will increase once these Data Deficient taxa are reassessed with more information to hand.

On a more positive note, approximately one third of endemics assessed are currently considered to be of Least Concern (LC) – i.e. they are not currently threatened on a global scale. Some of the endemics are widespread within Mozambique and can be locally abundant. For example, the strict-endemic *Grewia transzambesica* Wild (Malvaceae) has an extent of occurrence of c. 220,000 km^2^ and is frequent in the central lowlands of the country (Darbyshire et al. 2019). However, many of the LC species are much more range-restricted, but are not under threat owing to their habitat preferences. Many occur in rocky terrain and/or montane grasslands that are some of the least threatened habitats in Mozambique, due to a combination of remoteness, inaccessibility and limited agricultural value. A good example is the Chimanimani montane quartzite endemics, the majority of which are not significantly threatened (Timberlake et al. 2016b).

For the Mozambique flora as a whole, as of July 2019, 1,050 plant taxa (c. 17% of the total vascular flora) are listed on the IUCN Red List (https://www.iucnredlist.org). A total of 812 (77%) of these taxa are listed as LC, a much higher percentage than the equivalent for the endemics. Therefore, whilst there is still a long way to go before an exhaustive Red List can be achieved for Mozambique, the focus of the IUCN-SSC Southern African Plant Specialist Group on the endemic flora appears to be an effective strategy in identifying the taxa in most urgent need of conservation action.

## Conclusion: future priorities for the study of the endemic flora of Mozambique and its conservation

The checklist of endemic plants presented here provides a useful basis from which to build the evidence-base for effective conservation of the unique flora of Mozambique, for which the following next steps are underway:

• Complete the collation of existing data on endemic and near-endemic taxa, so that a detailed spatial analysis can be conducted to more accurately define Centres of Endemism and specific localities with concentrations of endemics. These results will allow for identification of critical knowledge gaps, and help effectively target sites for future field surveys.

• Complete a Red List of globally threatened species in Mozambique, with the eventual aims to assess the extinction risk for all endemic and near-endemic taxa, gather more information on species currently assessed as Data Deficient, and take active steps towards the conservation of all threatened species.

• Apply the accumulated plant distribution and Red List data, together with information on critical habitats, to identify and document Important Plant Areas. These data will also provide the botanical component for the identification of Key Biodiversity Areas.

Critical to the success of this work is the continued development of in-country capacity in field botany, taxonomy and conservation science in Mozambique, so that Mozambican practitioners are well placed to take forward the implementation of Mozambique’s commitments to protecting plant diversity under the CBD.

## Appendix 1

**Table:** Summary checklist of the endemic and near-endemic vascular plant taxa of Mozambique. Strict-endemic taxa are listed in bold. “Endemism” categories are abbreviated as follows: E = strict-endemic; NE1 = majority of range in Mozambique; NE2 = global range < 10,000 km^2^, NE3 = taxon known from five sites or fewer. Under “Life form”, (a) = annual; (c) = herbaceous climbing perennial; (e) = epiphytic perennial; (geo) = geophyte; (gram-a) = annual graminoid; (gram-p) = perennial graminoid; (p) = perennial (terrestrial, non-succulent); (par) = parasitic perennial; (s) = succulent. Under “Type only”, Y = known only from the type specimen or type locality. “Provinces” of Mozambique are abbreviated as follows CD = Cabo Delgado; G = Gaza; In = Inhambane; Mc = Manica; Mp = Maputo; Na = Nampula; Ni = Niassa; S = Sofala; T = Tete; Z = Zambezia. Under “Other Countries”, provinces of South Africa are abbreviated as follows: KN = KwaZulu Natal; LP = Limpopo; MP = Mpumalanga. Centres of Endemism (“CoE”) are coded as follows: 1 = Rovuma; 2 = Maputaland sensu lato; 2a = Maputaland sensu stricto; 2b = Lebombo Mountains (Sub-) Centre; 2c = Inhambane (Sub-) Centre; 3 = [Eastern] Afromontane sensu lato; 3a = Chimanimani-Nyanga (Sub-) Centre; 3b = Mulanje-Namuli-Ribaue (Sub-) Centre. See Materials and methods section for further explanation.

Family	Taxon	Endemism	Life form	Type only	Provinces	Other countries	CoE
** Pteridophyta **							
Pteridaceae	*Adiantum mendoncae* Alston	NE1	fern		Mc, Na, S	Zimbabwe	
**gymnospermae**							
Zamiaceae	*Encephalartos aplanatus* Vorster	NE2+3	cycad		Mp	eSwatini	2b
Zamiaceae	*Encephalartos chimanimaniensis* R.A.Dyer & I.Verd.	NE2+3	cycad		Mc	Zimbabwe	3a
** Zamiaceae **	**Encephalartos ferox G.Bertol subsp. emersus P.Rousseau, Vorster & A.E.van Wyk**	E	cycad		In		2c
Zamiaceae	Encephalartos ferox G.Bertol subsp. ferox	NE1	cycad		G, In, Mp	South Africa KN	2a, 2c
Zamiaceae	*Encephalartos gratus* Prain	NE1	cycad		Z	Malawi	3b
** Zamiaceae **	***Encephalartos munchii* R.A.Dyer & I.Verd.**	E	cycad		Mc		3a
Zamiaceae	*Encephalartos ngoyanus* I.Verd.	NE2	cycad		Mp	eSwatini, South Africa KN	2b
** Zamiaceae **	***Encephalartos pterogonus* R.A.Dyer & I.Verd.**	E	cycad		Mc		3a
Zamiaceae	*Encephalartos senticosus* Vorster		cycad		Mp	eSwatini, South Africa KN, MP	2b
** Zamiaceae **	***Encephalartos turneri* Lavranos & D.L.Goode**	E	cycad		Na, Ni		
Zamiaceae	*Encephalartos umbeluziensis* R.A.Dyer	NE2	cycad		Mp	eSwatini	2b
**Angiospermae: Magnoliids**							
** Annonaceae **	***Hexalobus mossambicensis* N.Robson**	E	shrub, tree		CD, Na		1
** Annonaceae **	***Huberantha mossambicensis* (Vollesen) Chaowasku**	E	shrub		Z		1
Annonaceae	*Monanthotaxis maputensis* P.H.Hoekstra	NE1	shrub, liana		G, In, Mp	South Africa KN	2a, 2c
Annonaceae	*Monanthotaxis suffruticosa* P.H.Hoekstra INED.	NE1+3	shrub		CD, Na	Tanzania	1
Annonaceae	*Monanthotaxis trichantha* (Diels) Verdc.	NE1	shrub		CD, Na	Tanzania	1
Annonaceae	*Monodora carolinae* Couvreur	NE2+3	shrub, tree		CD	Tanzania	1
Annonaceae	*Monodora stenopetala* Oliv.	NE1	shrub, tree		S, T	Malawi	
** Annonaceae **	***Uvaria rovumae* Deroin & Lötter**	E	liana		CD		1
Annonaceae	*Xylopia lukei* D.M.Johnson & Goyder	NE2	shrub, tree		CD	Tanzania	1
** Annonaceae **	***Xylopia tenuipetala* D.M.Johnson & Goyder**	E	shrub, tree		CD		1
** Annonaceae **	***Xylopia torrei* N.Robson**	E	shrub		G, In, Mp, Na		2a, 2c
**Angiospermae: Monocots**							
Amaryllidaceae	*Cryptostephanus vansonii* I.Verd.	NE1	herb (geo)		Mc, S, Z	Zimbabwe	3a, 3b
Amaryllidaceae	*Tulbaghia friesii* Suess.	NE2+3	herb (geo)		Mc	Zimbabwe	3a
Araceae	*Stylochaeton euryphyllum* Mildbr.	NE1	herb (geo)		CD, Na	Tanzania	1
Araceae	Stylochaeton natalense Schott subsp. maximum (Engl.) Bogner & Haigh	NE3	herb (geo)		Mp	Tanzania	
** Araceae **	***Stylochaeton tortispathum* Bogner & Haigh**	E	herb (geo)	Y	CD		1
Arecaceae	*Raphia australis* Oberm. & Strey	NE1	tree		Mp	South Africa KN	2a
Asparagaceae	*Asparagus chimanimanensis* Sebsebe		shrub		Mc	Zimbabwe	3a
Asparagaceae	*Asparagus petersianus* Kunth	NE1	shrub		CD, S, Z	Tanzania	
Asparagaceae	*Asparagus radiatus* Sebsebe	NE2+3	shrub		Mp	eSwatini	2b
Asparagaceae	Chlorophytum pygmaeum (Weim.) Kativu subsp. rhodesianum (Rendle) Kativu	NE2	herb (geo)		Mc	Zimbabwe	3a
Asparagaceae	*Dracaena pedicellata* (la Croix) Byng & Christenh. (=*Sansevieria pedicellata* la Croix)	NE1	herb (s)		Mc	Zimbabwe	3a
** Asparagaceae **	***Dracaena subspicata* (Baker) Byng & Christenh. (=*Sansevieria subspicata* Baker)**	E	herb (s)		Mp, Na, S, Z		
Asparagaceae	Eriospermum mackenii (Hook.f.) Baker subsp. phippsii (Wild) P.L.Perry	NE2+3	herb (geo)		Mc	Zimbabwe	3a
** Asphodelaceae **	***Aloe argentifolia* T.A.McCoy, Rulkens & O.J.Baptista**	E	shrub (s)		CD		
Asphodeleaceae	Aloe ballii Reynolds var. makurupiniensis Ellert	NE2	herb (s)		Mc	Zimbabwe	3a
** Asphodelaceae **	***Aloe cannellii* L.C.Leach**	E	herb (s)		Mc		3a
** Asphodelaceae **	***Aloe decurva* Reynolds**	E	herb (s)		Mc		3a
Asphodeleaceae	Aloe excelsa A.Berger var. breviflora L.C.Leach	NE2	tree (s)		Na, Z	Malawi	
Asphodeleaceae	Aloe hazeliana Reynolds var. hazeliana	NE2	herb (s)		Mc	Zimbabwe	3a
Asphodeleaceae	Aloe hazeliana Reynolds var. howmanii (Reynolds) S.Carter	NE2	herb (s)		Mc	Zimbabwe	3a
Asphodeleaceae	Aloe inyangensis Christian var. kimberleyana S.Carter	NE2	herb (s)		Mc	Zimbabwe	3a
Asphodeleaceae	Aloe marlothii A.Berger subsp. orientalis Glen & D.S.Hardy	NE1	tree (s)		G, Mp, S	eSwatini, South Africa KN	2a, 2c
Asphodeleaceae	*Aloe mawii* Christian	NE1	tree (s)		CD, Na, Ni, Z	Malawi, Tanzania	
** Asphodelaceae **	**Aloe menyharthii Baker subsp. ensifolia S.Carter**	E	herb (s)		Na, Z		
** Asphodelaceae **	***Aloe mossurilensis* Ellert**	E	herb (s)		Na		1
Asphodeleaceae	*Aloe munchii* Christian	NE2	shrub (s), tree (s)		Mc	Zimbabwe	3a
Asphodeleaceae	*Aloe plowesii* Reynolds	NE2	herb (s)		Mc	Zimbabwe	3a
** Asphodelaceae **	***Aloe ribauensis* T.A.McCoy, Rulkens & O.J.Baptista**	E	herb (s)		CD, Na		
** Asphodelaceae **	***Aloe rulkensii* T.A.McCoy & O.J.Baptista**	E	herb (s)		Na		3b
Asphodeleaceae	*Aloe suffulta* Reynolds	NE1	herb (s)		Mp	Malawi, Zimbabwe, South Africa KN	
** Asphodelaceae **	***Aloe torrei* I.Verd. & Christian**	E	herb (s)		Z		3b
Asphodeleaceae	*Aloe wildii* (Reynolds) Reynolds	NE2	herb (s)		Mc	Zimbabwe	3a
Asphodeleaceae	*Aloidendron tongaense* (Van Jaarsv.) Klopper & Gideon F.Sm. (=*Aloe tongaensis* Van Jaarsv.)	NE1	tree (s)		In, Mp, S	South Africa KN	
Commelinaceae	*Aneilema arenicola* Faden	NE2	herb (a)		Mp	South Africa KN	2a
** Commelinaceae **	***Aneilema mossambicense* (Faden) Faden INED. (=A. dregeanum Kunth subsp. mossambicense Faden)**	E	herb (p)		Na, Z		1
Commelinaceae	*Cyanotis chimanimaniensis* Faden INED.	NE2+3	herb (p)		Mc	Zimbabwe	3a
** Commelinaceae **	***Cyanotis namuliensis* Faden INED.**	E	herb (p)		Z		3b
Commelinaceae	*Triceratella drummondii* Brenan	NE3	herb (a)		Z	Zimbabwe	
Cymodoceaceae	*Thalassodendron leptocaule* Maria C.Duarte Bandeira & Romeiras	NE2	herb (seagrass)		Mp	South Africa KN	
Cyperaceae	*Cyperus longispicula* Muasya & D.A.Simpson	NE3	herb (gram-p)		Mc	Zimbabwe	
** Eriocaulaceae **	***Eriocaulon infaustum* N.E.Br.**	E	herb (a)	Y	Z		
Eriocaulaceae	*Eriocaulon mulanjeanum* S.M.Phillips	NE1+3	herb (a)		Z	Malawi	3b
Eriocaulaceae	*Mesanthemum africanum* Moldenke	NE2	herb (p)		Mc	Zimbabwe	3a
** Hydrocharitaceae **	**Halophila ovalis (R.Br.) Hook.f. subsp. linearis (Hartog) Hartog**	E	herb (seagrass)		In, Mp		
Iridaceae	*Dierama inyangense* Hilliard	NE2+3	herb (geo)		Mc	Zimbabwe	3a
Iridaceae	*Dierama plowesii* Hilliard	NE2+3	herb (geo)		Mc	Zimbabwe	3a
** Iridaceae **	**Freesia grandiflora (Baker) Klatt subsp. occulta J.C.Manning & Goldblatt**	E	herb (geo)	Y	Z		
Iridaceae	*Gladiolus brachyphyllus* F.Bolus	NE2	herb (geo)		Mp	eSwatini, South Africa MP	2b
Iridaceae	*Gladiolus zimbabweensis* Goldblatt	NE1	herb (geo)		Mc, Z	Zimbabwe	3a
Iridaceae	*Hesperantha ballii* Wild	NE2+3	herb (geo)		Mc	Zimbabwe	3a
** Iridaceae **	***Moraea niassensis* Goldblatt & J.C.Manning**	E	herb (geo)	Y	Ni		
** Iridaceae **	***Tritonia moggii* Oberm.**	E	herb (geo)		G, In, Mp, Z		2a, 2c
Orchidaceae	*Bonatea pulchella* Summerh.	NE1+3	herb (geo)		Mp	South Africa KN, LP, MP	2a
Orchidaceae	*Bulbophyllum ballii* P.J.Cribb	NE1	herb (e)		Mc, Na, Z	Zimbabwe	3a, 3b
Orchidaceae	*Cynorkis anisoloba* Summerh.	NE2	herb (geo)		Mc, S	Zimbabwe	3a
** Orchidaceae **	***Cyrtorchis glaucifolia* Summerh.**	E	herb (e)		Na		
Orchidaceae	*Disa chimanimaniensis* (H.P.Linder) H.P.Linder	NE2+3	herb (geo)		Mc	Zimbabwe	3a
Orchidaceae	*Disa zimbabweensis* H.P.Linder	NE2+3	herb (geo)		Mc	Zimbabwe	3a
** Orchidaceae **	***Disperis mozambicensis* Schltr.**	E	herb (geo)	Y	S		
** Orchidaceae **	***Eulophia biloba* Schltr.**	E	herb (?geo)	Y	S		
** Orchidaceae **	***Eulophia bisaccata* Kraenzl.**	E	herb (geo)	Y			
** Orchidaceae **	***Habenaria hirsutissima* Summerh.**	E	herb (geo)		Na, Ni		
** Orchidaceae **	***Habenaria mosambicensis* Schltr.**	E	herb (?geo)	Y	S		
Orchidaceae	Habenaria stylites Rchb.f. & S.Moore subsp. johnsonii (Rolfe) Summerh.	NE2+3	herb (geo)		Ni	Tanzania	
** Orchidaceae **	***Liparis hemipilioides* Schltr.**	E	herb (geo)	Y	S		
Orchidaceae	*Neobolusia ciliata* Summerh.	NE2+3	herb (geo)		Mc	Zimbabwe	3a
Orchidaceae	*Oeceoclades perrieri* (Schltr.) Garay & P.Taylor	NE3	herb (p)		Mp, S	Madagascar	
Orchidaceae	*Oligophyton drummondii* H.P.Linder & G.Will.	NE2+3	herb (geo)		Mc	Zimbabwe	3a
Orchidaceae	*Polystachya songaniensis* G.Will.	NE2	herb (e)		Z	Malawi	3b
Orchidaceae	*Polystachya subumbellata* P.J.Cribb & Podz.	NE2	herb (e)		Mc, S	Zimbabwe	3a
Orchidaceae	*Polystachya valentina* la Croix & P.J.Cribb	NE1	herb (p)		Mc, Z	Zimbabwe	3a, 3b
Orchidaceae	*Satyrium flavum* la Croix	NE2+3	herb (geo)		Mc	Zimbabwe	3a
Orchidaceae	*Schizochilus lepidus* Summerh.	NE2+3	herb (geo)		Mc	Zimbabwe	3a
** Poaceae **	***Alloeochaete namuliensis* Chippind.**	E	herb (gram-p)		Z		3b
** Poaceae **	***Baptorhachis foliacea* (Clayton) Clayton**	E	herb (gram-a)	Y	Na		3b
Poaceae	*Brachychloa fragilis* S.M.Phillips	NE2	herb (gram-a)		Mp	South Africa KN, LP	2a
Poaceae	*Brachychloa schiemanniana* (Schweick.) S.M.Phillips	NE1	herb (gram-p)		G, In, Mp	South Africa KN	2a, 2c
Poaceae	*Danthoniopsis chimanimaniensis* (J.B.Phipps) Clayton	NE2+3	herb (gram-p)		Mc	Zimbabwe	3a
** Poaceae **	***Digitaria appropinquata* Goetgh.**	E	herb (gram-a)	Y	Z		3b
** Poaceae **	***Digitaria fuscopilosa* Goetgh.**	E	herb (gram-p)	Y	Mc		3a
** Poaceae **	***Digitaria megasthenes* Goetgh.**	E	herb (gram-p)		Ni, Z		
Poaceae	*Eragrostis desolata* Launert	NE2+3	herb (gram-p)		Mc	Zimbabwe	3a
Poaceae	*Eragrostis moggii* De Winter	NE1	herb (gram-p)		In, Mp	South Africa KN	2a, 2c
** Poaceae **	***Eragrostis sericata* Cope**	E	herb (gram-p)		In		2c
Poaceae	*Eriochloa rovumensis* (Pilg.) Clayton	NE1	herb (gram-a)		Na, Ni, Z	Tanzania	
** Poaceae **	***Trichoneura schlechteri* Ekman**	E	herb (gram-p)		In, Mp		2a, 2c
Restionaceae	*Platycaulos quartziticola* (H.P.Linder) H.P.Linder & C.R.Hardy	NE2+3	herb (gram-p)		Mc	Zimbabwe	3a
Velloziaceae	*Xerophyta argentea* (Wild) L.B.Sm. & Ayensu	NE2+3	shrub, herb (p)		Mc	Zimbabwe	3a
Velloziaceae	*Xerophyta kirkii* (Hemsl.) L.B.Sm. & Ayensu	NE1	shrub, tree		Ni, Z	Malawi	3b
Velloziaceae	*Xerophyta pseudopinifolia* Behnke	NE1	shrub		Na, Ni, Z	Malawi	
Velloziaceae	*Xerophyta splendens* (Rendle) N.L.Menezes	NE2+3	shrub, tree		Z	Malawi	3b
Xyridaceae	*Xyris asterotricha* Lock	NE2+3	herb (p)		Mc	Zimbabwe	3a
Xyridaceae	*Xyris makuensis* N.E.Br.	NE2	herb (p)		Z	Malawi	3b
** Zingiberaceae **	***Siphonochilus kilimanensis* (Gagnep.) B.L.Burtt**	E	herb (geo)		Na, S, Z		
**Angiospermae: Eu-Dicots**							
Acanthaceae	*Barleria delagoensis* Oberm.	NE1	herb (p), shrub		G, In, Mp, S	South Africa KN	
Acanthaceae	*Barleria fissimuroides* I.Darbysh.	NE2+3	shrub		Mc	Zimbabwe	3a
Acanthaceae	Barleria fulvostellata C.B.Clarke subsp. mangochiensis I.Darbysh.	NE2+3	herb (p), shrub		Ni	Malawi	
Acanthaceae	*Barleria laceratiflora* Lindau	NE2+3	herb (p)		Na	Tanzania	1
Acanthaceae	*Barleria oxyphylla* Lindau	NE2	herb (p)		Mp	eSwatini, South Africa MP	
** Acanthaceae **	***Barleria setosa* (Klotzsch) I.Darbysh.**	E	herb (p), shrub		Na		1
** Acanthaceae **	***Barleria torrei* I.Darbysh.**	E	shrub		Ni		
Acanthaceae	*Barleria vollesenii* I.Darbysh.	NE2+3	herb (p)		Ni	Tanzania	
** Acanthaceae **	***Blepharis dunensis* Vollesen**	E	herb (p)		Na, Z		1
** Acanthaceae **	***Blepharis gazensis* Vollesen**	E	herb (p)		G, S		
Acanthaceae	*Blepharis swaziensis* Vollesen	NE2	herb (p)		Mp	eSwatini, South Africa KN	2b
Acanthaceae	*Blepharis torrei* Vollesen	NE2+3	herb (p)		Ni	Tanzania	
Acanthaceae	*Cephalophis lukei* Vollesen	NE3	herb (p)		S	Kenya	
Acanthaceae	*Dicliptera quintasii* Lindau	NE2	herb (p)		Mp	South Africa KN	2a
** Acanthaceae **	***Duosperma dichotomum* Vollesen**	E	herb (p), shrub		CD		1
Acanthaceae	*Ecbolium glabratum* Vollesen	NE1	herb (p)		G, Mp	eSwatini, South Africa KN, MP	2a, 2b
** Acanthaceae **	***Ecbolium hastatum* Vollesen**	E	herb (p), shrub		G, In, Mp		2a, 2c
** Acanthaceae **	***Isoglossa namuliensis* I.Darbysh. & T.Harris**	E	herb (p)	Y	Z		3b
Acanthaceae	*Justicia attenuifolia* Vollesen	NE1	herb (p)		Ni	Tanzania	
** Acanthaceae **	***Justicia gorongozana* Vollesen**	E	herb (p)		CD, S		
** Acanthaceae **	***Justicia niassensis* Vollesen**	E	shrub		CD, Na		1
Acanthaceae	*Justicia subcordatifolia* Vollesen & I.Darbysh. (=*J. hedrenii* Vollesen)	NE2	herb (p)		Mc	Zimbabwe	3a
Acanthaceae	*Lepidagathis plantaginea* Mildbr.	NE1	herb (p)		CD, Na	Tanzania	1
Acanthaceae	*Sclerochiton apiculatus* Vollesen	NE1+2	shrub		Mp	South Africa KN	2a
Acanthaceae	*Sclerochiton coeruleus* (Lindau) S.Moore	NE1	shrub		G, In, Mc, Na, Z	Zimbabwe	
** Acanthaceae **	***Sclerochiton hirsutus* Vollesen**	E	shrub		Z		3b
** Aizoaceae **	***Trianthema mozambiquense* H.E.K.Hartmann & Liede**	E	herb	Y	Mp		2a
Amaranthaceae	*Caroxylon littoralis* (Moq.) Akhani & Roalson	NE2	herb (p), shrub		In	Madagascar, Europa Is.	
** Amaranthaceae **	***Celosia nervosa* C.C.Towns.**	E	herb		In, Mp, Na		
** Amaranthaceae **	***Celosia pandurata* Baker**	E	herb		S, Z		
** Amaranthaceae **	***Salicornia mossambicensis* (Brenan) Piirainen & G.Kadereit**	E	herb (p)		In		2c
** Anacardiaceae **	***Ozoroa gomesiana* R.Fern. & A.Fern.**	E	shrub, tree		In		2c
Anacardiaceae	Ozoroa obovata (Oliv.) R.Fern. & A.Fern. var. elliptica R.Fern. & A.Fern.	NE1	shrub, tree		G, In, Mc, Mp, S, T, Z	Zimbabwe	
Anacardiaceae	*Rhus acuminatissima* R.Fern. & A.Fern. (=*Searsia acuminatissima* (R.Fern. & A.Fern.) Moffett)	NE1	shrub, tree		Na, Z	Malawi	
Apiaceae	*Afrosciadium rhodesicum* (Cannon) P.J.D.Winter	NE2	herb (p)		Mc	Zimbabwe	3a
** Apiaceae **	***Centella obtriangularis* Cannon**	E	herb (p)		Mc		3a
Apiaceae	*Pimpinella mulanjensis* C.C.Towns.	NE2+3	herb (p)		Z	Malawi	3b
Apocynaceae	Asclepias cucullata (Schltr.) Schltr. subsp. scabrifolia (S.Moore) Goyder	NE2+3	herb (geo)		Mc	Zimbabwe	3a
Apocynaceae	*Asclepias graminifolia* (Wild) Goyder	NE2+3	herb (geo)		Mc	Zimbabwe	3a
Apocynaceae	*Aspidoglossum glabellum* Kupicha	NE2+3	herb (geo)		Mc	Zimbabwe	3a
Apocynaceae	*Aspidoglossum hirundo* Kupicha	NE1	herb (geo)		Na, Z	Zimbabwe	
** Apocynaceae **	***Ceropegia aloicola* M.G.Gilbert INED.**	E	herb (s)		Mp		2b
Apocynaceae	*Ceropegia chimanimaniensis* M.G.Gilbert INED.	NE2+3	herb (geo)		Mc	Zimbabwe	3a
** Apocynaceae **	***Ceropegia cyperifolia* Bruyns**	E	herb (geo)		Ni		
** Apocynaceae **	***Ceropegia gracilidens* Bruyns**	E	herb (geo)		CD, Na, Z		
Apocynaceae	*Ceropegia monteiroae* Hook.f.	NE1	herb (s)		In, Mp	South Africa KN	2a, 2c
** Apocynaceae **	***Ceropegia muchevensis* M.G.Gilbert INED.**	E	herb (s)		S		
** Apocynaceae **	***Ceropegia nutans* (Bruyns) Bruyns**	E	herb (geo)		Z		3b
Apocynaceae	*Ceropegia vahrmeijeri* (R.A.Dyer) Bruyns	NE1+2	herb (geo)		Mp	South Africa KN	2a
** Apocynaceae **	***Cynanchum oresbium* (Bruyns) Goyder**	E	herb (s)		Na		
** Apocynaceae **	***Emicocarpus fissifolius* K.Schum.& Schltr.**	E	herb (p)		Mp		2a
** Apocynaceae **	***Huernia erectiloba* L.C.Leach & Lavranos**	E	shrub (s)		CD, Na, Z		
Apocynaceae	*Huernia leachii* Lavranos	NE1+2	herb (s)		Mc	Malawi	
** Apocynaceae **	**Huernia verekeri Stent subsp. pauciflora (L.C.Leach) Bruyns**	E	shrub (s)		In, S		
Apocynaceae	Huernia volkartii Werderm. & Peitsch. var. repens (Lavranos) Lavranos	NE2+3	herb (s)		Mc	Zimbabwe	
Apocynaceae	*Marsdenia cynanchoides* Schltr.	NE1	liana		CD, S, Z	Tanzania, Zimbabwe	
Apocynaceae	*Marsdenia gazensis* S.Moore	NE2	liana		Mc	Zimbabwe	3a
** Apocynaceae **	***Orbea halipedicola* L.C.Leach**	E	shrub (s)		S		
Apocynaceae	*Orbea longidens* (N.E.Br.) L.C.Leach	NE1+2	herb (s)		Mp	South Africa KN	2a
Apocynaceae	Pachycarpus concolor E.Mey. subsp. arenicola Goyder	NE2	herb (geo)		Mp	South Africa KN	2a
Apocynaceae	*Raphionacme pulchella* Venter & R.L.Verh.	NE3	herb (geo)		Mc	Zimbabwe	3a
Apocynaceae	*Secamone delagoensis* Schltr.	NE1	liana		G, In, Mp	South Africa KN	2a, 2c
Apocynaceae	*Stapelia unicornis* C.A.Luckh.	NE2	herb (s)		Mp	eSwatini, South Africa KN	2b
** Apocynaceae **	***Stomatostemma pendulina* Venter & D.V.Field (=*Cryptolepis pendulin*a (Venter & D.V.Field) P.I.Forst.**)	E	shrub		Na, Z		
Araliaceae	*Cussonia arenicola* Strey	NE1	shrub		In, Mp	South Africa KN	2a, 2c
** Asteraceae **	***Adelostigma athrixioides* Steetz [uncertain species**]	E	herb		In		2c
Asteraceae	*Anisopappus paucidentatus* Wild	NE2	herb (p)		Mc	Zimbabwe	3a
Asteraceae	*Aster chimanimaniensis* W.Lippert (=*Afroaster chimanimaniensis* (W.Lippert) J.C.Manning & Goldblatt)	NE2	herb (p)		Mc	Zimbabwe	3a
** Asteraceae **	***Bothriocline moramballae* (Oliv. & Hiern) O.Hoffm.**	E	herb (s), shrub (s)		Na, Z		3b
** Asteraceae **	***Bothriocline steetziana* Wild & G.V.Pope**	E	herb (a)		In, Na, Z		
Asteraceae	*Chrysocoma mozambicensis* Ehr.Bayer	NE1	shrub		In, Mp	South Africa KN, MP	2a, 2c
Asteraceae	*Cineraria pulchra* Cron	NE2	herb (p), shrub		Mc, S	Zimbabwe	3a
Asteraceae	*Distephanus inhacensis* (G.V.Pope) R.G.C.Boon & Glen	NE1	shrub, liana		G, In, Mp	South Africa KN	2a, 2c
Asteraceae	*Gutenbergia westii* (Wild) Wild & G.V.Pope	NE1+2	herb (p)		Mc	Zimbabwe	3a
** Asteraceae **	***Gyrodoma hispida* (Vatke) Wild**	E	herb (a)		G, In, Mp, S, Z		
Asteraceae	*Helichrysum acervatum* S.Moore	NE2	herb (p)		Mc	Zimbabwe	3a
Asteraceae	*Helichrysum africanum* (S.Moore) Wild (=*Calomeria africana* (S.Moore) Heine)	NE2+3	herb (p), shrub		Mc	Zimbabwe	3a
Asteraceae	*Helichrysum chasei* Wild	NE2	herb (p)		Mc	Zimbabwe	3a
Asteraceae	*Helichrysum lastii* Engl.	NE2	herb (p), shrub		Z	Malawi	3b
** Asteraceae **	***Helichrysum moggii* Wild**	E	herb (p)		Mp		2a
Asteraceae	*Helichrysum moorei* Staner	NE2+3	herb (p)		Mc	Zimbabwe	3a
Asteraceae	*Helichrysum rhodellum* Wild	NE2+3	herb (p)		Mc	Zimbabwe	3a
Asteraceae	*Helichrysum silvaticum* Hilliard		herb (p)		G, In, Mp	South Africa KN	2a, 2c
Asteraceae	*Kleinia chimanimaniensis* van Jaarsv.	NE2+3	herb (s), shrub (s)		Mc	Zimbabwe	3a
Asteraceae	*Lopholaena brickellioides* S.Moore	NE2+3	shrub, tree		Mc	Zimbabwe	3a
Asteraceae	*Schistostephium oxylobum* S.Moore	NE2	herb (p), shrub		Mc	Zimbabwe	3a
Asteraceae	*Senecio aetfatensis* B.Nord.	NE2+3	herb (p)		Mc	Zimbabwe	3a
** Asteraceae **	***Senecio forbesii* Oliv. & Hiern [uncertain species**]	E	herb	Y	Unknown		
Asteraceae	*Senecio peltophorus* Brenan	NE2+3	herb (p)		Z	Malawi	3b
Asteraceae	Vernonia calvoana (Hook.f.) Hook.f. subsp. meridionalis (Wild) C.Jeffrey (=*Baccharoides calvoana* (Hook.f.) Isawumi, El-Ghazaly & B.Nord. subsp. meridionalis (Wild) Isuwami, El-Ghazaly & B.Nord.)	NE2	herb (p), shrub		Mc, S	Zimbabwe	3a
Asteraceae	Vernonia muelleri Wild subsp. muelleri	NE2	shrub		Mc	Zimbabwe	3a
Asteraceae	*Vernonia nepetifolia* Wild	NE2+3	shrub		Mc	Zimbabwe	3a
Balsaminaceae	*Impatiens psychadelphoides* Launert	NE1	herb (p)		Mc, Z	Zimbabwe	3a, 3b
Balsaminaceae	*Impatiens salpinx* G.M.Schulze & Launert	NE2	herb (p)		Mc	Zimbabwe	3a
** Balsaminaceae **	***Impatiens wuerstenii* S.B.Janssens & Dessein**	E	herb (p)		S		3a
** Bignoniaceae **	***Dolichandrone alba* (Sim) Sprague**	E	shrub, tree		G, In, Mp		2a, 2c
** Boraginaceae **	***Cordia mandimbana* E.S.Martins**	E	tree	Y	Ni		
** Boraginaceae **	***Cordia megiae* J.E.Burrows**	E	tree		S		
** Boraginaceae **	***Cordia stuhlmannii* Gürke**	E	shrub, tree		S, Z		
Burseraceae	*Commiphora mombassensis* Engl.	NE3	shrub, tree		CD	Tanzania	1
Burseraceae	*Commiphora schlechteri* Engl.	NE1	shrub, tree		G, In, Mp	South Africa KN, ?Zimbabwe	2a, 2c
Campanulaceae	*Lobelia blantyrensis* E.Wimm.	NE2	herb (a), herb (p)		Z	Malawi	3b
Campanulaceae	*Lobelia cobaltica* S.Moore	NE2+3	herb (a), herb (p)		Mc	Zimbabwe	3a
Campanulaceae	Wahlenbergia subaphylla (Baker) Thulin subsp. scoparia (Wild) Thulin	NE2+3	herb (p)		Mc	Zimbabwe	3a
Capparaceae	Capparis viminea Hook.f. & Thomson ex Oliv. var. orthacantha (Gilg & Gilg-Ben.) DeWolf	NE2+3	shrub		CD	Tanzania	1
Capparaceae	*Maerua acuminata* Oliv.	NE1	shrub, tree		CD	Tanzania	1
** Capparaceae **	***Maerua andradae* Wild**	E	herb (p), shrub		CD		1
** Capparaceae **	***Maerua brunnescens* Wild**	E	shrub		In, Mc, Mp, S, T, Z		
** Capparaceae **	***Maerua scandens* (Klotzsch) Müll.Berol. ex B.D.Jacks.**	E	shrub, liana		G, Na, T, Z		
Capparaceae	*Maerua schliebenii* Gilg-Ben.	NE1	shrub		Na	Tanzania	1
** Caprifoliaceae **	***Pterocephalus centennii* M.J.Cannon**	E	shrub	Y	Mc		3a
** Caryophyllaceae **	***Dianthus chimanimaniensis* S.S.Hooper**	E	herb	Y	Mc		3a
** Celastraceae **	***Crossopetalum mossambicense* I.Darbysh.**	E	shrub		CD		1
** Celastraceae **	***Elaeodendron fruticosum* N.Robson**	E	shrub, tree		G, In		2a, 2c
Celastraceae	*Gymnosporia arenicola* Jordaan	NE1	shrub, tree		G, In, Mp, S, Z	South Africa KN	
** Celastraceae **	***Gymnosporia gurueensis* (N.Robson) Jordaan**	E	shrub, tree		Z		
Celastraceae	*Gymnosporia markwardii* Jordaan	NE1	shrub		In, Mp, Z	South Africa KN	
Celastraceae	*Gymnosporia oxycarpa* (N.Robson) Jordaan	NE2	shrub		G	South Africa LP	
Celastraceae	*Maytenus chasei* N.Robson	NE1	shrub, tree		Mc, Z	Zimbabwe	3a, 3b
Celastraceae	Prionostemma delagoensis (Loes.) N.Hallé var. delagoensis (=*Hippocratea delagoensis* Loes.)	NE1	shrub, liana		G, Mp	South Africa KN	2a, 2c
Celastraceae	*Salacia orientalis* N.Robson	NE1	shrub, liana		CD	Tanzania	1
Chrysobalanaceae	*Maranthes goetzeniana* (Engl.) Prance	NE1	tree		Mc, Na, S, Z	Zimbabwe, Tanzania	3a, 3b
Cleomaceae	*Cleome bororensis* (Klotzsch) Oliv. (=*Sieruela bororensis* (Klotzsch) Roalson & J.C.Hall)	NE1	herb (a)		G, Mp, S, Z	Tanzania, South Africa KN	
Clusiaceae	*Garcinia acutifolia* N.Robson	NE1	shrub, tree		CD	Tanzania	1
Combretaceae	*Combretum andradae* Exell & J.G.García	NE1	shrub, liana		CD, Na, Ni	Tanzania	
** Combretaceae **	***Combretum caudatisepalum* Exell & J.G.García**	E	shrub		CD, Na		1
** Combretaceae **	***Combretum lasiocarpum* Engl. & Diels**	E	shrub, tree		Na, T, Z		
Combretaceae	*Combretum lindense* Exell & Mildbr.	NE2+3	shrub, liana		CD	Tanzania	1
** Combretaceae **	***Combretum stocksii* Sprague**	E	shrub		CD		1
** Combretaceae **	***Terminalia barbosae* (Exell) Gere & Boatwr. (=*Pteleopsis barbosae* Exell)**	E	tree		CD, Na		1
** Convolvulaceae **	***Ipomoea ephemera* Verdc.**	E	herb (a)		Na, Z		
** Convolvulaceae **	**Ipomoea venosa (Desr.) Roem. & Schult. subsp. stellaris (Baker) Verdc. var. obtusifolia Verdc.**	E	herb (p)	Y	Mp		2a
** Convolvulaceae **	***Turbina longiflora* Verdc.**	E	herb (c)		CD, In, Mp		
** Crassulaceae **	***Crassula leachii* R.Fern.**	E	herb (p)		Mc		
Crassulaceae	*Crassula maputensis* R.Fern.	NE1	herb (a), herb (p)		Mp	South Africa KN	2a
** Crassulaceae **	***Crassula morrumbalensis* R.Fern.**	E	herb (p)	Y	Z		
Crassulaceae	*Crassula zombensis* Baker f.	NE2+3	herb (p)		Z	Malawi	3b
Crassulaceae	*Kalanchoe elizae* A.Berger	NE1	herb (s)		Na, Ni, Z	Malawi	
** Crassulaceae **	***Kalanchoe fernandesii* Raym.-Hamet**	E	herb (p)	Y	Na		1
** Crassulaceae **	***Kalanchoe hametiorum* Raym.-Hamet**	E	herb (p)		CD, Na, Z		
Crassulaceae	Kalanchoe velutina Welw. ex Britten subsp. chimanimaniensis (R.Fern.) R.Fern.	NE2+3	herb (s)		Mc	Zimbabwe	3a
Cucurbitaceae	*Eureiandra eburnea* C.Jeffrey	NE1	herb (c)		T	Zambia, Zimbabwe	
Cucurbitaceae	*Momordica henriquesii* Cogn.	NE1	herb (c)		CD, Na	Tanzania	1
** Cucurbitaceae **	***Momordica mossambica* H.Schaef.**	E	liana	Y	Na		1
Dichapetalaceae	*Dichapetalum barbosae* Torre	NE1	shrub, liana		CD, S, Z	Tanzania	
Dichapetalaceae	*Dichapetalum deflexum* (Klotzsch) Engl.	NE1	shrub		CD, In, Na	Tanzania	
Dichapetalaceae	*Dichapetalum macrocarpum* Engl.	NE1	shrub		CD	Tanzania	1
Dilleniaceae	*Tetracera bussei* Gilg	NE3	shrub		Ni	Tanzania	
Ebenaceae	*Diospyros rotundifolia* Hiern	NE1	tree		G, In, Mp	South Africa KN	2a, 2c
Ebenaceae	Euclea racemosa L. subsp. sinuata F.White	NE1	shrub, tree		In, Mp	South Africa KN	2a, 2c
Ericaceae	*Erica lanceolifera* S.Moore	NE2	shrub		Mc	Zimbabwe	3a
Ericaceae	Erica pleiotricha S.Moore var. blaerioides (Wild) R.Ross	NE2	shrub		Mc	Zimbabwe	3a
Ericaceae	Erica pleiotricha S.Moore var. pleiotricha	NE2+3	shrub		Mc	Zimbabwe	3a
Ericaceae	*Erica wildii* Brenan	NE2+3	herb (p), shrub		Mc	Zimbabwe	3a
Erythroxylaceae	*Nectaropetalum carvalhoi* Engl.	NE1+2	shrub, tree		CD, Na	Tanzania? - see note in F.T.E.A. Erythroxylaceae: 8 (1984)	1
** Euphorbiaceae **	***Croton aceroides* Radcl.-Sm.**	E	tree		In		2c
** Euphorbiaceae **	***Croton inhambanensis* Radcl.-Sm.**	E	shrub, tree		In		2c
Euphorbiaceae	*Croton kilwae* Radcl.-Sm.	NE1	shrub		CD, Na	Tanzania	1
** Euphorbiaceae **	**Croton leuconeurus Pax subsp. mossambicensis Radcl.-Sm.**	E	shrub, tree		S, Z		
Euphorbiaceae	*Crotonogynopsis australis* Kenfack & Gereau	NE2	tree		Z	Tanzania	
Euphorbiaceae	*Erythrococca zambesiaca* Prain	NE2	shrub		S	Malawi	
** Euphorbiaceae **	**Euphorbia ambroseae L.C.Leach var. ambrosae**	E	shrub (s)		In, S, Z		
Euphorbiaceae	Euphorbia ambroseae L.C.Leach var. spinosa L.C.Leach	NE1	shrub (s)		In, S, T	Malawi	
** Euphorbiaceae **	***Euphorbia angularis* Klotzsch**	E	shrub (s)		CD, Na		1
** Euphorbiaceae **	***Euphorbia baylissii* L.C.Leach**	E	shrub (s)		G, In, Mp		2a, 2c
** Euphorbiaceae **	***Euphorbia bougheyi* L.C.Leach**	E	tree (s)		CD, In, S, Z		
Euphorbiaceae	*Euphorbia citrina* S.Carter	NE2	shrub		Mc, S	Zimbabwe	3b
** Euphorbiaceae **	***Euphorbia contorta* L.C.Leach**	E	shrub (s)		Na, Ni, Z		
** Euphorbiaceae **	***Euphorbia corniculata* R.A.Dyer**	E	shrub (s)		CD, Na, Ni		
Euphorbiaceae	*Euphorbia crebrifolia* S.Carter	NE2	herb (p)		Mc	Zimbabwe	3a
** Euphorbiaceae **	***Euphorbia crenata* (N.E.Br.) Bruyns (=*Monadenium crenatum* N.E.Br.)**	E	herb (p)	Y	Mc		
Euphorbiaceae	*Euphorbia decliviticola* L.C.Leach	NE1+2+3	shrub (s), tree (s)		Na, Z	Malawi	3b
Euphorbiaceae	Euphorbia depauperata A.Rich. var. tsetserrensis S.Carter	NE2+3	herb (p)		Mc	Zimbabwe	3a
Euphorbiaceae	Euphorbia grandicornis Blanc subsp. grandicornis	NE1	shrub (s)		G, Mc, Mp	eSwatini, South Africa KN	
** Euphorbiaceae **	**Euphorbia grandicornis Blanc subsp. sejuncta L.C.Leach**	E	shrub (s)		Na		
** Euphorbiaceae **	***Euphorbia graniticola* L.C.Leach**	E	shrub (s), tree (s)		Mc		3a
Euphorbiaceae	*Euphorbia keithii* R.A.Dyer	NE2+3	shrub (s), tree (s)		Mp	eSwatini	2b
** Euphorbiaceae **	**Euphorbia knuthii Pax subsp. johnsonii (N.E.Br.) L.C.Leach**	E	shrub (s)		Mp, S		
Euphorbiaceae	Euphorbia knuthii Pax subsp. knuthii	NE1	shrub (s)		Mp	eSwatini, South Africa KN, MP	2a, 2b
** Euphorbiaceae **	***Euphorbia marrupana* Bruyns**	E	shrub (s)		Ni		
Euphorbiaceae	*Euphorbia mlanjeana* L.C.Leach	NE1+3	shrub (s)		Na, Ni, Z	Malawi	
** Euphorbiaceae **	***Euphorbia namuliensis* Bruyns**	E	shrub (s)		Z		3b
** Euphorbiaceae **	***Euphorbia neohalipedicola* Bruyns (=*Synadenium halipedicola* L.C.Leach)**	E	shrub	Y	S		
Euphorbiaceae	*Euphorbia neorugosa* Bruyns nom. inval. (=*Monadenium rugosum* S.Carter)	NE2+3	herb (p)		CD	Tanzania	1
** Euphorbiaceae **	***Euphorbia plenispina* S.Carter**	E	shrub (s)		Mc		
** Euphorbiaceae **	***Euphorbia ramulosa* L.C.Leach**	E	shrub (s)		Na, Ni, Z		
** Euphorbiaceae **	***Euphorbia schlechteri* Pax**	E	herb (p)		G, Mp		2a
** Euphorbiaceae **	***Euphorbia stenocaulis* Bruyns**	E	shrub (s)	Y	Z		
Euphorbiaceae	*Euphorbia torrei* (L.C.Leach) Bruyns	NE1	shrub		CD	Tanzania	1
** Euphorbiaceae **	***Euphorbia unicornis* R.A.Dyer**	E	shrub (s)		CD		
** Euphorbiaceae **	**Jatropha latifolia Pax var. subeglandulosa Radcl.-Sm.**	E	herb (p)	Y	Mp		2b
** Euphorbiaceae **	***Jatropha scaposa* Radcl.-Sm.**	E	herb (p)		Mp, Na, S		
** Euphorbiaceae **	***Jatropha subaequiloba* Radcl.-Sm.**	E	shrub		In		2c
Euphorbiaceae	Mallotus oppositifolius (Geiseler) Müll.Arg. var. lindicus (Radcl.-Sm.) Radcl.-Sm.	NE1	tree		CD, Na	Tanzania	1
** Euphorbiaceae **	**Tragia glabrata (Müll.Arg.) Pax & K.Hoffm. var. hispida Radcl.-Sm.**	E	herb (c)	Y	Mp		2a
** Euphorbiaceae **	**Tragia shirensis Prain var. glabriuscula Radcl.-Sm.**	E	herb (p)	Y	Na		
Euphorbiaceae	Tragia shirensis Prain var. shirensis	NE1	herb (p)		Z	Malawi	
** Fabaceae **	***Acacia latispina* J.E.Burrows & S.M.Burrows (=*Vachellia latispina* (J.E.Burrows & S.M.Burrows) Kyal. & Boatwr.)**	E	tree		CD		1
Fabaceae	*Acacia latistipulata* Harms (=*Senegalia latistipulata* (Harms) Kyal. & Boatwr.)	NE1	shrub		CD, Na, T	Tanzania	
** Fabaceae **	***Acacia quiterajoensis* Timberlake & Lötter**	E	shrub, tree		CD		1
** Fabaceae **	***Acacia torrei* Brenan (=*Vachellia torrei* (Brenan) Kyal. & Boatwr.)**	E	shrub		S		
** Fabaceae **	***Adenopodia schlechteri* (Harms) Brenan**	E	liana, shrub		G, Mp		2a
Fabaceae	*Aeschynomene aphylla* Wild	NE2+3	shrub		Mc	Zimbabwe	3a
Fabaceae	*Aeschynomene chimanimaniensis* Verdc.	NE2+3	shrub		Mc	Zimbabwe	3a
Fabaceae	*Aeschynomene grandistipulata* Harms	NE2+3	shrub		Mc	Zimbabwe	3a
Fabaceae	*Aeschynomene inyangensis* Wild	NE2+3	shrub		Mc	Zimbabwe	3a
** Fabaceae **	**Aeschynomene minutiflora Taub. subsp. grandiflora Verdc.**	E	herb (a)		Na, Z		
** Fabaceae **	**Aeschynomene mossambicensis Verdc. subsp. mossambicensis**	E	herb (a), herb (p)		Na, Z		
Fabaceae	*Aeschynomene pawekiae* Verdc.	NE2+3	herb (p)		Ni	Malawi	
Fabaceae	*Baphia macrocalyx* Harms	NE1	tree		CD	Tanzania	1
** Fabaceae **	**Baphia massaiensis Taub. subsp. gomesii (Baker f.) Brummitt**	E	shrub, tree		CD, In, Na, Ni	Tanzania?	
** Fabaceae **	***Baphia ovata* Sim (=Baphia kirkii Baker subsp. ovata (Sim) Soladoye)**	E	shrub, tree		G, In		2c
** Fabaceae **	**Baphia punctulata Harms subsp. palmensis Soladoye**	E	shrub, tree	Y	CD		1
** Fabaceae **	***Bauhinia burrowsii* E.J.D.Schmidt**	E	shrub		In		2c
Fabaceae	*Berlinia orientalis* Brenan	NE1	tree		CD	Tanzania	1
** Fabaceae **	***Brachystegia oblonga* Sim**	E	tree		Na, Z		1
** Fabaceae **	***Bussea xylocarpa* (Sprague) Sprague & Craib**	E	tree		Mc		
** Fabaceae **	***Chamaecrista paralias* (Brenan) Lock**	E	herb (p), shrub, tree		In, Na		
Fabaceae	*Crotalaria assurgens* Polhill	NE3	herb (p)		Ni	Tanzania	
Fabaceae	Crotalaria dura J.M.Wood & M.S.Evans subsp. mozambica Polhill	NE1	herb (p), shrub		G, In, Mp	South Africa KN	2a, 2c
Fabaceae	*Crotalaria insignis* Polhill	NE2	shrub		Mc	Zimbabwe	3a
Fabaceae	Crotalaria lanceolata E.Mey. subsp. exigua Polhill	NE1	herb (a), herb (p)		Na, Z	Malawi	
** Fabaceae **	***Crotalaria misella* Polhill**	E	herb (a)		CD	Tanzania?	1
** Fabaceae **	***Crotalaria mocubensis* Polhill**	E	herb (a)		S, T, Z		
** Fabaceae **	***Crotalaria namuliensis* Polhill & T.Harris**	E	herb (a), herb (p)		Z		3b
** Fabaceae **	***Crotalaria paraspartea* Polhill**	E	herb (a)		Na		
Fabaceae	*Crotalaria phylicoides* Wild	NE2+3	herb (p), shrub		Mc	Zimbabwe	3a
Fabaceae	*Crotalaria schlechteri* Baker f.	NE1+2	herb (p)		G, Mp	South Africa MP	2a
Fabaceae	*Crotalaria schliebenii* Polhill	NE1+2+3	herb (a), herb (p)		Na	Tanzania	1
** Fabaceae **	***Crotalaria torrei* Polhill**	E	shrub		Z		3b
Fabaceae	*Dialium schlechteri* Harms	NE1	tree		G, In, Mp	South Africa KN	2a, 2c
Fabaceae	Dichrostachys cinerea (L.) Wight & Arn. subsp. africana Brenan & Brummitt var. pubescens Brenan & Brummitt	NE1	shrub, tree		G, Mc, S	Zimbabwe	
** Fabaceae **	***Entada mossambicensis* Torre**	E	shrub		Na		
Fabaceae	*Entada stuhlmannii* (Taub.) Harms	NE1	liana		CD, Na, Z	Tanzania	1
Fabaceae	*Gelrebia rostrata* (N.E.Br.) Gagnon & G.P.Lewis (=*Caesalpinia rostrata* N.E.Br.)	NE2+3	shrub, liana		Mp	South Africa MP	2a, 2b
** Fabaceae **	***Guibourtia sousae* J.Leonard**	E	tree	Y	In		2c
** Fabaceae **	***Icuria dunensis* Wieringa**	E	tree		Na, Z		1
Fabaceae	*Indigofera cecilii* N.E.Br.	NE1	herb (p), shrub		Mc, S	Zimbabwe	3a
Fabaceae	*Indigofera concinna* Baker	NE1	herb (a)		CD, Na	Tanzania	1
** Fabaceae **	***Indigofera emarginella* A.Rich. var. *marrupaënsis* Schrire**	E	shrub	Y	Ni		
Fabaceae	Indigofera erythrogramma Baker subsp. nampulensis Schrire	NE1+3	herb (a)		Na	Malawi	
** Fabaceae **	***Indigofera gobensis* Schrire**	E	herb (p)		Mp		2b
Fabaceae	*Indigofera graniticola* J.B.Gillett	NE2+3	herb (a)		Na	Tanzania	1
** Fabaceae **	***Indigofera mendoncae* J.B.Gillett**	E	herb (p)		G, In		2c
** Fabaceae **	***Indigofera namuliensis* Schrire**	E	herb (a)		Z		3b
Fabaceae	Indigofera nyassica Gilli var. brevior (J.B.Gillett) J.B.Gillett	NE3	herb (a), herb (p)		Ni	Tanzania	
Fabaceae	*Indigofera podophylla* Harv.	NE1	herb (p)		G, In, Mp	South Africa KN	2a, 2c
** Fabaceae **	***Indigofera pseudomoniliformis* Schrire**	E	shrub		Na, Ni, Z		
** Fabaceae **	***Indigofera torrei* J.B.Gillett**	E	herb (p), shrub		G		
Fabaceae	Indigofera vicioides Jaub. & Spach subsp. excelsa Schrire	NE2+3	herb (p), shrub		Mc	Zimbabwe	3a
Fabaceae	*Lotus wildii* J.B.Gillett	NE2	herb (p), shrub		S	Zimbabwe	3a
** Fabaceae **	***Macrotyloma decipiens* Verdc.**	E	herb	Y	Na		1
** Fabaceae **	***Micklethwaitia carvalhoi* (Harms) G.P.Lewis & Schrire**	E	tree		CD, Na		1
** Fabaceae **	***Millettia ebenifera* (Bertol.) J.E.Burrows & Lötter**	E	shrub, tree		G, In		2c
Fabaceae	*Millettia makondensis* Harms	NE1	shrub		CD	Tanzania	1
** Fabaceae **	***Millettia mossambicensis* J.B.Gillett**	E	tree		Na, S		
Fabaceae	*Mimosa mossambicensis* Brenan	NE1	shrub, liana		S, T	Malawi	
Fabaceae	*Ormocarpum schliebenii* Harms	NE1	shrub		CD, Na	Tanzania	1
Fabaceae	Otholobium foliosum (Oliv.) C.H.Stirt. subsp. gazense (Baker f.) Verdc.	NE2+3	shrub		Mc	Zimbabwe	3a
Fabaceae	*Pearsonia mesopontica* Polhill	NE2+3	herb (p)		Mc	Zimbabwe	3a
Fabaceae	*Rhynchosia chimanimaniensis* Verdc.	NE2+3	herb (p), shrub		Mc	Zimbabwe	3a
** Fabaceae **	**Rhynchosia clivorum S.Moore subsp. gurueensis Verdc.**	E	herb (p), shrub	Y	Z		3b
Fabaceae	*Rhynchosia genistoides* Burtt Davy	NE2+3	shrub		Mp	South Africa MP	2b
Fabaceae	*Rhynchosia stipata* Meikle	NE2+3	herb (c), herb (p)		Mc	Zimbabwe	3a
Fabaceae	*Rhynchosia swynnertonii* Baker f.	NE2	herb (c), liana		Mc	Zimbabwe	3a
** Fabaceae **	***Rhynchosia torrei* Verdc.**	E	shrub		Z		3b
** Fabaceae **	***Scorodophloeus torrei* Lock**	E	shrub, tree		Na, Z		1
Fabaceae	*Sphenostylis zimbabweensis* Mithen	NE3	herb (c), herb (p)		Mc	Zimbabwe	3a
Fabaceae	*Tephrosia chimanimaniana* Brummitt	NE1+2+3	shrub		Mc	Zimbabwe	3a
** Fabaceae **	***Tephrosia faulknerae* Brummitt**	E	shrub		Na, Z		
Fabaceae	Tephrosia forbesii Baker subsp. forbesii	NE1	herb (p)		G, Mp	South Africa KN	2a
** Fabaceae **	**Tephrosia forbesii Baker subsp. inhacensis Brummitt**	E	herb (p)		Mp		2a
Fabaceae	*Tephrosia gobensis* Brummitt	NE2+3	shrub		Mp	eSwatini	2b
Fabaceae	Tephrosia longipes Meisn. var. drummondii (Brummitt) Brummitt	NE2+3	herb (p)		Mc	Zimbabwe	3a
Fabaceae	Tephrosia longipes Meisn. var. swynnertonii (Baker f.) Brummitt	NE2	herb (p), shrub		Mc	Zimbabwe	3a
** Fabaceae **	***Tephrosia miranda* Brummitt**	E	shrub		Na		
Fabaceae	*Tephrosia montana* Brummitt	NE2	shrub		Mc, S	Zimbabwe	3a
Fabaceae	*Tephrosia praecana* Brummitt	NE2	shrub, tree		Mc	Zimbabwe	3a
** Fabaceae **	**Tephrosia reptans Baker var. microfoliata (Pires da Lima) Brummitt**	E	herb (a)		CD, Na, Z		1
** Fabaceae **	**Tephrosia whyteana Baker f. subsp. gemina Brummitt**	E	shrub		Z		3b
** Fabaceae **	***Xylia mendoncae* Torre**	E	shrub, tree		In		2c
Gentianaceae	*Exacum zombense* N.E.Br.	NE1	herb (a)		Mc, Na, Ni, Z	Malawi	3a, 3b
** Gentianaceae **	***Faroa involucrata* (Klotzsch) Knobl.**	E	herb (a)		Na, Z		
Geraniaceae	*Geranium exellii* J.R.Laundon	NE2+3	herb (p)		Mc	Zimbabwe	3a
Geraniaceae	*Pelargonium mossambicense* Engl.	NE2	herb (p)		S	Zimbabwe	3a
** Gesneriaceae **	***Streptocarpus acicularis* I.Darbysh. & Massingue**	E	herb (p)	Y	Mc		3a
** Gesneriaceae **	***Streptocarpus brachynema* Hilliard & B.L.Burtt**	E	herb		S		3a
Gesneriaceae	*Streptocarpus erubescens* Hilliard & B.L.Burtt	NE2	herb		Ni	Malawi	3b
Gesneriaceae	Streptocarpus grandis N.E.Br. subsp. septentrionalis Hilliard & B.L.Burtt	NE2	herb		Mc	Zimbabwe	3a
Gesneriaceae	*Streptocarpus hirticapsa* B.L.Burtt	NE2	herb (p)		Mc	Zimbabwe	3a
Gesneriaceae	*Streptocarpus leptopus* Hilliard & B.L.Burtt	NE2	herb (p)		Z	Malawi	3b
Gesneriaceae	*Streptocarpus michelmorei* B.L.Burtt	NE2	herb (p)		Mc, S?	Zimbabwe	3a
Gesneriaceae	*Streptocarpus milanjianus* Hilliard & B.L.Burtt	NE2+3	herb (p)		Z	Malawi	3b
** Gesneriaceae **	***Streptocarpus montis-bingae* Hilliard & B.L.Burtt**	E	herb (p)	Y	Mc		3a
** Gesneriaceae **	***Streptocarpus myoporoides* Hilliard & B.L.Burtt**	E	herb (p)		Na		3b
Gesneriaceae	*Streptocarpus umtaliensis* B.L.Burtt	NE2	herb		Mc	Zimbabwe	3a
** Lamiaceae **	***Acrotome mozambiquensis* G.Taylor**	E	herb (p)		Mp		2a
Lamiaceae	*Aeollanthus viscosus* Ryding	NE2+3	shrub		Mc	Zimbabwe	3a
** Lamiaceae **	***Clerodendrum abilioi* R.Fern.**	E	herb (p)	Y	Na		1
** Lamiaceae **	**Clerodendrum cephalanthum Oliv. subsp. cephalanthum var. torrei R.Fern.**	E	liana, shrub	Y?	CD		1
Lamiaceae	*Clerodendrum lutambense* Verdc.	NE1+3	shrub		CD	Tanzania	1
** Lamiaceae **	**Clerodendrum robustum Klotzsch var. macrocalyx R.Fern.**	E	herb (p)	Y	Mc		
Lamiaceae	*Coleus caudatus* (S.Moore) E.Downes & I.Darbysh. (=*Plectranthus caudatus* S.Moore)	NE2+3	herb (p)		Mc	Zimbabwe	3a
** Lamiaceae **	***Coleus cucullatus* (A.J.Paton) A.J.Paton (=*Plectranthus cucullatus* A.J.Paton)**	E	herb (p), shrub		Na		3b
** Lamiaceae **	***Coleus namuliensis* E.Downes & I.Darbysh.**	E	herb (p)		Z		3b
Lamiaceae	*Coleus psammophilus* (Codd) A.J.Paton (=*Plectranthus psammophilus* Codd)	NE1	herb (p)		In, Mp	South Africa KN	2a, 2c
Lamiaceae	*Coleus sessilifolius* (A.J.Paton) A.J.Paton (=*Plectranthus sessilifolius* A.J.Paton)	NE2	herb (p)		Mc	Zimbabwe	3a
** Lamiaceae **	**Leucas nyassae Gürke var. velutina (C.H.Wright ex Baker) Sebald**	E	herb (p)		Ni		
Lamiaceae	*Ocimum natalense* Ayob. ex A.J. Paton	NE2	herb (p), shrub		G, Mp	South Africa KN	2a
Lamiaceae	*Ocimum reclinatum* (S.D.Will. & K.Balkwill) A.J.Paton	NE2	herb (p)		Mp	South Africa KN	2a
Lamiaceae	*Orthosiphon scedastophyllus* A.J.Paton	NE2+3	herb (p)		CD	Tanzania	1
Lamiaceae	*Plectranthus chimanimanensis* S.Moore	NE1	herb (p), shrub		Mc, S	Zimbabwe	3a
** Lamiaceae **	***Plectranthus guruensis* A.J.Paton**	E	herb (p)		Z		3b
Lamiaceae	*Plectranthus mandalensis* Baker	NE2	herb (a), herb (p)		Z	Malawi	3b
Lamiaceae	*Premna hans-joachimii* Verdc.	NE2	shrub		CD	Tanzania	1
Lamiaceae	*Premna tanganyikensis* Moldenke	NE1	shrub, tree		CD, Na	Tanzania	1
** Lamiaceae **	**Rotheca luembensis (De Wild.) R.Fern. subsp. niassensis (R.Fern.) R.Fern.**	E	herb (p)		Ni		
** Lamiaceae **	**Rotheca sansibarensis (Gürke) Steane & Mabb. subsp. sansibarensis var. eratensis (R.Fern.) R.Fern.**	E	shrub	Y	Na		
Lamiaceae	*Rotheca teaguei* (Hutch.) R.Fern.	NE2+3	herb (p)		Mc	Zimbabwe	
Lamiaceae	*Rotheca verdcourtii* (R.Fern.) R.Fern.	NE2	shrub, tree		Mc	Zimbabwe	3a
Lamiaceae	*Stachys didymantha* Brenan	NE2	herb (p)		Z	Malawi	3b
Lamiaceae	*Syncolostemon flabellifolius* (S.Moore) A.J.Paton	NE2+3	shrub, tree		Mc	Zimbabwe	3a
Lamiaceae	*Syncolostemon namapaensis* D.F.Otieno	NE2+3	herb (p)		Na	Tanzania	
Lamiaceae	*Syncolostemon oritrephes* (Wild) D.F.Otieno	NE2+3	herb (p), shrub		Mc	Zimbabwe	3a
Lamiaceae	*Vitex carvalhi* Gürke	NE1	shrub, tree		CD, Na	Tanzania	1
Lamiaceae	*Vitex mossambicensis* Gürke	NE1	tree		CD, Na	Tanzania	1
Lentibulariaceae	*Utricularia podadena* P.Taylor	NE2+3	herb (p)		Ni	Malawi	
** Linaceae **	***Hugonia elliptica* N.Robson**	E	shrub, liana		Z		1
Linaceae	*Hugonia grandiflora* N.Robson	NE3	shrub, tree, liana		CD	Tanzania	1
Linderniaceae	*Crepidorhopalon flavus* (S.Moore) I.Darbysh. & Eb.Fisch. (=*Lindernia flava* S.Moore)	NE2	herb (p)		Mc	Zimbabwe	3a
** Linderniaceae **	***Crepidorhopalon namuliensis* I.Darbysh. & Eb.Fisch.**	E	herb (p)		Z		3b
** Loranthaceae **	***Agelanthus deltae* (Baker & Sprague) Polhill & Wiens**	E	shrub (par)		S, T, Z		
Loranthaceae	*Agelanthus igneus* (Danser) Polhill & Wiens	NE1+3	shrub (par)		CD, S, T, Z	Tanzania	
Loranthaceae	*Agelanthus patelii* Polhill & Timberlake INED.	NE2+3	shrub (par)		Z	Malawi	3b
Loranthaceae	*Englerina oedostemon* (Danser) Polhill & Wiens	NE2	shrub (par)		Mc	Zimbabwe	3a
** Loranthaceae **	***Englerina schlechteri* (Engl.) Polhill & Wiens**	E	shrub (par)		G, In, Mp		2a, 2c
Loranthaceae	*Englerina swynnertonii* (Sprague) Polhill & Wiens	NE2+3	shrub (par)		Mc	Zimbabwe	3a
Loranthaceae	*Englerina triplinervia* (Baker & Sprague) Polhill & Wiens	NE3	shrub (par)		CD, Na	Tanzania	1
** Loranthaceae **	***Helixanthera schizocalyx* T.Harris, I.Darbysh. & Polhill**	E	shrub (par)		Z		3b
** Lythraceae **	***Ammannia elata* R.Fern.**	E	herb (a)	Y	Z		
** Lythraceae **	***Ammannia fernandesiana* S.A.Graham & Gandhi**	E	herb (p)		In, S		
** Lythraceae **	***Ammannia gazensis* (A.Fern.) S.A.Graham & Gandhi**	E	herb (a)	Y	G		
Lythraceae	*Ammannia linearis* (Hiern) S.A.Graham & Gandhi	NE1	herb (a)		Na, S, Z	Tanzania	
** Lythraceae **	***Ammannia moggii* (A.Fern.) S.A.Graham & Gandhi**	E	herb (p)	Y	Na		1
Lythraceae	*Ammannia mossambicensis* (A.Fern. & Diniz) S.A.Graham & Gandhi	NE3	herb (a), herb (p)		Na	Tanzania, Zimbabwe	
** Lythraceae **	***Ammannia parvula* S.A.Graham & Gandhi**	E	herb (a)		Na		
** Lythraceae **	***Ammannia pedroi* (A.Fern. & Diniz) S.A.Graham & Gandhi**	E	herb (a)		CD, Na		1
** Lythraceae **	***Ammannia polycephala* (Peter) S.A.Graham & Gandhi**	E	herb (p)		S		
** Lythraceae **	***Ammannia ramosissima* (A.Fern. & Diniz) S.A.Graham & Gandhi**	E	herb (a)	Y	Ni	?Malawi	
** Lythraceae **	***Ammannia spathulata* (A.Fern.) S.A.Graham & Gandhi**	E	herb (p)	Y	S		
Malpighiaceae	Acridocarpus natalitius A.Juss. var. linearifolius Launert	NE1	shrub, tree, liana		In, Mp	eSwatini, South Africa KN	2a
Malpighiaceae	Triaspis hypericoides (DC.) Burch. subsp. canescens (Engl.) Immelman	NE2	shrub		Mp	South Africa MP	2b
** Malpighiaceae **	***Triaspis suffulta* Launert**	E	liana		In		2c
** Malvaceae **	***Cola cheringoma* Cheek**	E	tree		S		
** Malvaceae **	***Cola clavata* Mast.**	E	tree		S, Z		
Malvaceae	*Cola mossambicensis* Wild	NE1	tree		Mc, Na, S, Z	Malawi, Tanzania	
Malvaceae	*Corchorus velutinus* Wild	NE1	shrub		G, In	Zimbabwe, South Africa LP	
** Malvaceae **	***Dombeya lastii* K.Schum.**	E	shrub		Z		3b
** Malvaceae **	***Dombeya leachii* Wild**	E	shrub		Na		3b
** Malvaceae **	***Eriolaena rulkensii* Dorr**	E	shrub, tree		CD		1
Malvaceae	*Glyphaea tomentosa* Mast.	NE1	shrub, tree		Na, S, Z	Malawi	
Malvaceae	*Grewia filipes* Burret	NE2+3	shrub, tree		CD	Tanzania	1
Malvaceae	*Grewia hornbyi* Wild	NE1	shrub		G, In, Mc, Mp, S, T	Zimbabwe, South Africa KN	
** Malvaceae **	***Grewia limae* Wild**	E	shrub, tree		CD		1
** Malvaceae **	**Grewia occidentalis L. var. littoralis Wild**	E	shrub		G, In, Mp		2a, 2c
** Malvaceae **	***Grewia transzambesica* Wild**	E	shrub, tree		CD, Na, S, Z		
Malvaceae	*Hermannia micropetala* Harv.	NE1	herb (p), shrub		G, In, Mp, S	South Africa KN	2a, 2c
** Malvaceae **	***Hermannia torrei* Wild**	E	herb (p), shrub	Y	G		2c
Malvaceae	*Hibiscus burtt-davyi* Dunkley	NE3	shrub, tree		Mc	Malawi, Zimbabwe	3a, 3b
** Malvaceae **	***Hibiscus rupicola* Exell**	E	herb (p), shrub	Y	T	?Malawi	
** Malvaceae **	***Hibiscus torrei* Baker f.**	E	herb (p), shrub	Y	Ni		
Malvaceae	*Microcos microthyrsa* (K.Schum. ex Burret) Burret (=*Grewia microthyrsa* K.Schum. ex Burret)	NE1	shrub		G, In, Mp	South Africa KN, LP	2a, 2b, 2c
** Malvaceae **	***Thespesia mossambicensis* (Exell & Hillc.) Fryxell**	E	shrub, tree		CD		1
Malvaceae	*Triumfetta kirkii* Mast.	NE1	herb (a)		CD, Na, S	Tanzania	
** Melastomataceae **	***Antherotoma angustifolia* (A.Fern. & R.Fern.) Jacq.-Fél.**	E	herb (p), shrub		CD, Na		1
Melastomataceae	Dissotis johnstoniana Baker f. var. johnstoniana (=Dissotidendron johnstonianum (Baker f.) Ver,-Lib. & G.Kadereit var. johnstonianum)	NE2+3	shrub		Z	Malawi	3b
Melastomataceae	*Dissotis pulchra* A.Fern. & R.Fern.	NE2+3	herb (p), shrub		Mc	Zimbabwe	3a
Melastomataceae	*Dissotis swynnertonii* (Baker f.) A.Fern. & R.Fern. (=*Pseudosbeckia swynnertonii* (Baker f.) A. Fern. & R.Fern.)	NE2+3	shrub		Mc	Zimbabwe	3a
** Melastomataceae **	***Memecylon aenigmaticum* R.D.Stone**	E	shrub	Y	CD		1
** Melastomataceae **	***Memecylon incisilobum* R.D.Stone & I.G.Mona**	E	tree		G		2a
** Melastomataceae **	***Memecylon insulare* A.Fern. & R.Fern.**	E	shrub		In		2c
Melastomataceae	*Memecylon nubigenum* R.D.Stone & I.G.Mona	NE1+2+3	tree		Na, Z	Malawi	3b
Melastomataceae	*Memecylon rovumense* R.D.Stone & I.G.Mona	NE2+3	shrub, tree		CD	Tanzania	1
** Melastomataceae **	***Memecylon torrei* A.Fern. & R.Fern.**	E	shrub, tree		CD, Na		1
** Melastomataceae **	***Warneckea albiflora* R.D.Stone & N.P.Tenza**	E	tree		CD		1
** Melastomataceae **	***Warneckea cordiformis* R.D.Stone**	E	shrub, tree		CD		1
Melastomataceae	*Warneckea parvifolia* R.D.Stone & Ntetha	NE2+3	shrub, tree		Mp	South Africa KN	2a
** Melastomataceae **	***Warneckea sessilicarpa* (A.Fern. & R.Fern.) Jacq.-Fel.**	E	shrub, tree		Na		1
Melastomataceae	*Warneckea sousae* (A.Fern. & R.Fern.) A.E.van Wyk	NE1	shrub, tree		CD, Na, S, Z	Tanzania	
Melianthaceae	*Bersama swynnertonii* Baker f.	NE2	shrub, tree		Mc	Zimbabwe	3a
Menispermaceae	*Albertisia delagoensis* (N.E.Br.) Forman	NE1	shrub, liana		In, Mp, Na, S, Z	South Africa KN	
Menispermaceae	*Cissampelos hirta* Klotzsch	NE1	liana		G, In, Mp	South Africa KN	2a, 2c
Menispermaceae	*Tinospora mossambicensis* Engl.	NE3	liana		Unknown	Tanzania	
Moraceae	*Bosqueiopsis carvalhoana* Engl.		shrub		CD, Na	Tanzania	1
** Moraceae **	***Dorstenia zambesiaca* Hijman**	E	herb (p)		Na, S		
** Moraceae **	***Ficus muelleriana* C.C.Berg**	E	shrub		Mc		3a
Myricaceae	*Myrica chimanimaniana* (Verdc. & Polhill) Christenh. & Byng (=*Morella chimanimaniana* Verdc.& Polhill)	NE2+3	shrub		Mc	Zimbabwe	3a
Myrtaceae	*Syzygium komatiense* Byng & Pahlad.	NE2+3	tree		Mp	South Africa MP	2b
Myrtaceae	*Syzygium niassense* Byng & J.E.Burrows	NE1	tree		CD, Na, Ni, S, Z	Tanzania?	
** Ochnaceae **	***Ochna angustata* N.Robson**	E	shrub, tree		CD, Na, S, Z		
** Ochnaceae **	***Ochna beirensis* N.Robson**	E	shrub, tree		S		
** Ochnaceae **	***Ochna dolicharthros* F.M.Crawford & I.Darbysh.**	E	shrub		CD		1
Oleaceae	*Olea chimanimani* Kupicha	NE2+3	shrub, tree		Mc	Zimbabwe	3a
Orobanchaceae	*Buchnera chimanimaniensis* Philcox	NE2	herb (a), herb (p)		Mc	Zimbabwe	3a
** Orobanchaceae **	***Buchnera namuliensis* Skan**	E	herb (a)		S, Z		
Orobanchaceae	*Buchnera subglabra* Philcox	NE2+3	herb (a)		Mc	Zimbabwe	3a
Orobanchaceae	*Buchnera wildii* Philcox	NE2	herb (a), herb (p)		Mc	Zimbabwe, ?Malawi	3a
** Orobanchaceae **	***Striga diversifolia* Pires de Lima**	E	herb (a)	Y	CD		1
Orobanchaceae	*Striga junodii* Schinz	NE1	herb (p)		In, Mp	South Africa KN, MP	2a, 2c
Passifloraceae	*Adenia dolichosiphon* Harms	NE1	herb (c)		CD, Mc, S, Z	Tanzania	
** Passifloraceae **	***Adenia mossambicensis* W.J.de Wilde**	E	herb (c)	Y	Na		
** Passifloraceae **	***Adenia zambesiensis* R.Fern. & A.Fern.**	E	herb (c)	Y	Z		
** Passifloraceae **	***Tricliceras auriculatum* (A.Fern. & R.Fern.) R.Fern.**	E	herb (a)		Na		
** Passifloraceae **	***Tricliceras elatum* (A.Fern. & R.Fern.) R.Fern.**	E	herb (a)		Na		
** Passifloraceae **	***Tricliceras lanceolatum* (A.Fern. & R.Fern.) R.Fern.**	E	herb (a)		Na, S		
** Passifloraceae **	**Tricliceras longepedunculatum (Mast.) R.Fern. var. eratense R.Fern.**	E	herb (p)		Na		
Penaeaceae	*Olinia chimanimani* T.Shah & I.Darbysh.	NE2+3	shrub, tree		Mc	Zimbabwe	3a
Peraceae	*Clutia sessilifolia* Radcl.-Sm.	NE2+3	shrub		Mc	Zimbabwe	3a
Phyllanthaceae	Phyllanthus bernierianus Müll.Arg. var. glaber Radcl.-Sm.	NE2+3	shrub		Mc	Zimbabwe	3a
** Phyllanthaceae **	***Phyllanthus manicaensis* Jean F.Brunel ex Radcl.-Sm.**	E	herb (p)		Mc	?Zimbabwe	3a
** Phyllanthaceae **	**Phyllanthus reticulatus Poir. var. orae-solis Radcl.-Sm.**	E	shrub, tree		Mp		2a
** Phyllanthaceae **	***Phyllanthus tsetserrae* Jean F.Brunel ex Radcl.-Sm.**	E	herb (p)	Y	Mc		3a
** Podostemaceae **	***Inversodicraea torrei* (C.Cusset) Cheek**	E	herb (p)		Z		3b
** Polygalaceae **	***Carpolobia suaveolens* Meikle**	E	shrub, tree		CD, In, Na, S, Z		
Polygalaceae	*Polygala adamsonii* Exell	NE2+3	herb (a)		Na, Z	Malawi	3b
** Polygalaceae **	***Polygala francisci* Exell**	E	herb (p), shrub		In, Mp	?Zimbabwe	2a, 2c
** Polygalaceae **	***Polygala limae* Exell**	E	herb (a)	Y	CD		1
** Polygalaceae **	***Polygala torrei* Exell**	E	herb (p)	Y	Mp		2a
Polygalaceae	*Polygala zambesiaca* Paiva	NE2	shrub		Mc	Zimbabwe	3a
Primulaceae	*Lysimachia gracilipes* (P.Taylor) U.Manns & Anderb.	NE2+3	herb (p)		S	Zimbabwe	3a
Proteaceae	*Faurea racemosa* Farmar	NE1+3	tree		Z	Malawi	3b
Proteaceae	*Faurea rubriflora* Marner	NE2	tree		Mc	Zimbabwe	3a
Proteaceae	*Leucospermum saxosum* S.Moore	NE3	shrub		Mc	Zimbabwe, South Africa LP MP	3
Proteaceae	Protea caffra Meisn. subsp. gazensis (Beard) Chisumpa & Brummitt	NE2	shrub, tree		Mc, S	Zimbabwe	3a
Proteaceae	*Protea enervis* Wild	NE2+3	herb (p)		Mc	Zimbabwe	3a
** Putranjivaceae **	**Drypetes gerrardii Hutch. var. angustifolia Radcl.-Sm.**	E	shrub, tree	Y	Mc		
Rhizophoraceae	*Cassipourea mossambicensis* (Brehmer) Alston	NE1	shrub, tree		CD, In, Mp	Tanzania, eSwatini, South Africa KN	
Rubiaceae	*Afrocanthium ngonii* (Bridson) Lantz	NE2	shrub, tree		Mc	Zimbabwe	3a
Rubiaceae	Afrocanthium racemulosum (S.Moore) Lantz var. nanguanum (Tennant) Bridson	NE1	shrub, tree		CD, Z	Tanzania	1
Rubiaceae	*Afrocanthium vollesenii* (Bridson) Lantz	NE3	shrub, tree		CD, Na	Tanzania	1
Rubiaceae	*Anthospermum ammanioides* S.Moore	NE1	shrub		Mc, S	Zimbabwe	3a
Rubiaceae	*Anthospermum vallicola* S.Moore	NE1	shrub		Mc, S	Zimbabwe	3a
Rubiaceae	*Anthospermum zimbabwense* Puff	NE2	shrub		Mc	Zimbabwe	3a
Rubiaceae	Canthium oligocarpum Hiern subsp. angustifolium Bridson	NE1	tree		Mc, S	Zimbabwe	3a
Rubiaceae	*Catunaregam stenocarpa* Bridson	NE1	shrub, tree		CD, Na, Ni, Z	Tanzania	
Rubiaceae	*Catunaregam swynnertonii* (S.Moore) Bridson	NE1	shrub, tree		CD, G, Mc, Na, S, T, Z	Zimbabwe	
** Rubiaceae **	***Chassalia colorata* J.E.Burrows**	E	shrub		CD		1
Rubiaceae	*Coffea salvatrix* Swynn. & Phillipson	NE1	shrub, tree		Mc, Z	Tanzania, Malawi, Zimbabwe	
Rubiaceae	*Coffea schliebenii* Bridson	NE2	shrub, tree		CD	Tanzania	1
** Rubiaceae **	***Conostomium gazense* Verdc.**	E	herb (p)	Y	G		2c
Rubiaceae	*Cuviera schliebenii* Verdc.	NE1	shrub, tree		CD, Na, Z	Tanzania	1
Rubiaceae	*Didymosalpinx callianthus* J.E.Burrows & S.M.Burrows	NE1+2+3	shrub		CD	Tanzania	1
Rubiaceae	*Empogona jenniferae* Cheek	NE2+3	tree		Mc	Zimbabwe	3a
Rubiaceae	*Empogona maputensis* (Bridson & A.E.van Wyk) Tosh & Robbr.	NE2+3	shrub		Mp	South Africa KN	2a
** Rubiaceae **	***Heinsia mozambicensis* (Verdc.) J.E.Burrows & S.M.Burrows**	E	shrub		Na		1
Rubiaceae	*Hymenodictyon austro-africanum* J.E.Burrows & S.M.Burrows	NE2	shrub, tree		G	South Africa LP	
Rubiaceae	*Hyperacanthus microphyllus* (K.Schum.) Bridson	NE1	shrub, tree		G, Mp, Na, S	South Africa KN, ?Zimbabwe	
Rubiaceae	*Leptactina papyrophloea* Verdc.	NE1+3	tree		CD	Tanzania	1
Rubiaceae	*Oldenlandia cana* Bremek.	NE2	herb (a)		Mc	Zimbabwe	3a
** Rubiaceae **	***Oldenlandia verrucitesta* Verdc.**	E	herb (a), herb (p)	Y	Z		
Rubiaceae	Otiophora inyangana N.E.Br. subsp. inyangana	NE1+2	herb (p), shrub		Mc	Zimbabwe	3a
Rubiaceae	Otiophora inyangana N.E.Br. subsp. parvifolia (Verdc.) Puff	NE1+2	herb (p), shrub		Mc	Zimbabwe	3a
Rubiaceae	*Otiophora lanceolata* Verdc.	NE1+2	herb (p), shrub		Mc	Zimbabwe	3a
Rubiaceae	*Oxyanthus biflorus* J.E.Burrows & S.M.Burrows	NE1+2+3	shrub		CD	Tanzania	1
Rubiaceae	*Oxyanthus latifolius* Sond.	NE1	tree		G, In, Mp, S, Z	South Africa KN	
Rubiaceae	*Oxyanthus strigosus* Bridson & J.E.Burrows	NE1+2	shrub		CD	Tanzania	1
Rubiaceae	*Pavetta chapmanii* Bridson	NE2	shrub, tree		Z	Malawi	3b
Rubiaceae	Pavetta comostyla S.Moore subsp. comostyla var. inyangensis (Bremek.) Bridson	NE1+2	shrub, tree		Mc, S	Zimbabwe	3a
** Rubiaceae **	***Pavetta curalicola* J.E.Burrows**	E	shrub		CD, Na		1
Rubiaceae	*Pavetta decumbens* K.Schum. & K.Krause	NE1	shrub		CD, Na, S, Z	Tanzania	1
** Rubiaceae **	***Pavetta dianeae* J.E.Burrows & S.M.Burrows**	E	shrub		CD, Na, Z		1
** Rubiaceae **	**Pavetta gardeniifolia A.Rich. var. appendiculata (De Wild.) Bridson**	E	shrub, tree		Ni, Z		
Rubiaceae	*Pavetta gracillima* S.Moore	NE1	shrub		In, Mc, S	Zimbabwe	
** Rubiaceae **	***Pavetta gurueensis* Bridson**	E	shrub		Z		3b
** Rubiaceae **	***Pavetta incana* Klotzsch**	E	shrub		T		
Rubiaceae	*Pavetta klotzschiana* K.Schum.	NE1	shrub		CD, In, Mc, Na, S, T, Z	Malawi, Zimbabwe	
Rubiaceae	*Pavetta lindina* Bremek.	NE1+2	shrub		CD	Tanzania	1
Rubiaceae	*Pavetta micropunctata* Bridson	NE1+2	shrub		Na	Tanzania	1
** Rubiaceae **	***Pavetta mocambicensis* Bremek.**	E	shrub		CD, Na		1
** Rubiaceae **	***Pavetta pumila* N.E.Br.**	E	shrub		S		
Rubiaceae	*Pavetta tendagurensis* Bremek.	NE1	shrub		CD, Na	Tanzania	1
Rubiaceae	*Pavetta umtalensis* Bremek.	NE1+2	shrub, tree		Mc	Zimbabwe	3a
Rubiaceae	*Pavetta vanwykiana* Bridson	NE2	shrub		Mp	South Africa KN	2a, 2b
Rubiaceae	Pentas zanzibarica (Klotzsch) Vatke subsp. milangiana (Verdc.) Verdc.	NE1	herb (p), shrub		Na, Z	Malawi	3b
** Rubiaceae **	***Polysphaeria harrisii* I.Darbysh. & C.Langa**	E	shrub		Z		3b
** Rubiaceae **	***Polysphaeria ribauensis* I. Darbysh. & C.Langa**	E	shrub		Na		3b
** Rubiaceae **	**Psychotria amboniana K.Schum. subsp. mosambicensis (E.M.A.Petit) Verdc.**	E	shrub		G, In, Mp		2a, 2c
Rubiaceae	*Psychotria angustibracteata* (Verdc.) J.E.Burrows	NE1	shrub, tree		Mc, Na, S, Z	Zimbabwe	3a, 3b
Rubiaceae	*Psydrax fragrantissimus* (K.Schum.) Bridson	NE1	shrub, tree		Mp	South Africa KN	2a
Rubiaceae	*Psydrax micans* (Bullock) Bridson	NE1	tree, liana		CD, Na, S	Tanzania	
** Rubiaceae **	***Psydrax moggii* Bridson**	E	shrub, tree		CD, G, In, Mp, Na, S		
Rubiaceae	*Pyrostria chapmanii* Bridson	NE1+2+3	shrub, tree		Na, Z	Malawi	3b
Rubiaceae	Rothmannia fischeri (K.Schum.) Bullock subsp. moramballae (Hiern) Bridson	NE1	tree		CD, In, Mc, Mp, S, Z	South Africa KN; Zimbabwe	
Rubiaceae	Rytigynia celastroides (Baill.) Verdc. var australis Verdc.	NE1	shrub		In, Mp	South Africa KN	2a, 2c
** Rubiaceae **	***Rytigynia torrei* Verdc.**	E	shrub		CD, Na		
Rubiaceae	*Sericanthe chimanimaniensis* Wursten & De Block INED.	NE1+2	shrub, tree		Mc	Zimbabwe	3a
** Rubiaceae **	***Spermacoce kirkii* (Hiern.) Verdc.**	E	herb (a), herb (p)		G, In, S, Z		
** Rubiaceae **	***Spermacoce schlechteri* K.Schum. ex Verdc.**	E	herb (p)		In, Na, S, Z	?Tanzania	
** Rubiaceae **	***Tarenna longipedicellata* (J.G.García) Bridson**	E	shrub		S, Z		
** Rubiaceae **	***Tarenna pembensis* J.E.Burrows**	E	tree		CD, Na		1
** Rubiaceae **	***Triainolepis sancta* Verdc.**	E	shrub		In		2c
Rubiaceae	Tricalysia coriacea (Benth.) Hiern subsp. angustifolia (J.G.Garcia) Robbr.	NE1	shrub, tree		Mc, S	Zimbabwe	3a
Rubiaceae	*Tricalysia ignota* Bridson	NE2+3	shrub, tree		Mc	Malawi; Zimbabwe	3a, 3b
** Rubiaceae **	**Tricalysia jasminiflora (Klotzsch) Benth. & Hook.f. ex Hiern var. hypotephros Brenan**	E	shrub, tree		Z		
Rubiaceae	*Tricalysia schliebenii* Robbr.	NE1	shrub		CD, Na, Z	Tanzania	1
Rubiaceae	*Tricalysia semidecidua* Bridson	NE1	shrub		CD	Tanzania	1
** Rubiaceae **	***Vangueria domatiosa* J.E.Burrows**	E	tree		CD		1
Rubiaceae	*Vangueria monteiroi* (Oliv.) Lantz (=*Lagynias monteiroi* (Oliv.) Bridson)	NE1	shrub, tree		G, Mp	eSwatini, South Africa KN	2a, 2c
** Rutaceae **	***Teclea crenulata* (Engl.) Engl. (=*Todallia crenulata* Engl.)**	E	unknown	Y	Z		
** Rutaceae **	***Vepris allenii* I.Verd.**	E	shrub, tree		CD	Possibly Tanzania	1
Rutaceae	*Vepris carringtoniana* Mendonça	NE1	shrub		In, Mp	eSwatini, South Africa KN, LP, MP	2a, 2b, 2c
Rutaceae	*Vepris drummondii* Mendonça	NE2+3	shrub		Mc	Zimbabwe	3a
** Rutaceae **	***Vepris macedoi* (Exell & Mendonça) Mziray**	E	tree		Na		3b
Rutaceae	*Vepris myrei* (Exell & Mendonça) Mziray	NE1	shrub, tree		In, S, T	Malawi, Zimbabwe	
** Rutaceae **	***Zanthoxylum delagoense* P.G.Waterman**	E	shrub, tree		G, In, Mp, S		2a, 2c
Rutaceae	*Zanthoxylum tenuipedicellatum* (Kokwaro) Vollesen	NE2+3	shrub, tree		Na	Tanzania	1
Santalaceae	*Thesium chimanimaniense* Brenan	NE2+3	herb (p)		Mc	Zimbabwe	3a
Santalaceae	*Thesium dolichomeres* Brenan	NE2+3	herb (p)		Mc	Zimbabwe	3a
** Santalaceae **	***Thesium inhambanense* Hilliard**	E	herb (p)	Y	In	Possibly Malawi	2c
Santalaceae	*Thesium pygmaeum* Hilliard	NE2+3	herb (p)		Mc	Zimbabwe	3a
Santalaceae	*Thesium vahrmeijeri* Brenan	NE1	herb (a)		In, Mp	South Africa KN	2a, 2c
** Santalaceae **	***Viscum littorum* Polhill & Wiens**	E	shrub		CD		1
** Sapindaceae **	***Allophylus mossambicensis* Exell**	E	shrub		G, In		2a, 2c
** Sapindaceae **	***Allophylus torrei* Exell & Mendonça**	E	shrub, tree		CD, Na		
Sapotaceae	*Synsepalum chimanimani* S.Rokni & I.Darbysh.	NE2+3	shrub, tree		Mc	Zimbabwe	3a
Sapotaceae	*Synsepalum muelleri* (Kupicha) T.D.Penn.	NE1	shrub, tree		Na, Z	Malawi	3b
Scrophulariaceae	*Jamesbrittenia carvalhoi* (Engl.) Hilliard	NE2	herb (p), shrub		Mc, S	Zimbabwe	3a
Scrophulariaceae	*Selago anatrichota* Hilliard	NE2+3	herb (p)		Mc	Zimbabwe	3a
Scrophulariaceae	Selago swynnertonii (S.Moore) Eyles var. leiophylla (Brenan) Hilliard	NE2	herb (p)		Mc	Zimbabwe	3a
** Solanaceae **	***Solanum litoraneum* A.E.Gonç.**	E	shrub		In, Mp		2a, 2c
Solanaceae	*Solanum torreanum* A.E.Gonç.	NE1	herb (c)		Mp	eSwatini, South Africa KN MP	2a
Thymelaeaceae	*Gnidia chapmanii* B.Peterson	NE2+3	shrub		Z	Malawi	3b
Thymelaeaceae	*Struthiola montana* B.Peterson	NE2+3	shrub		Mc	Zimbabwe	3a
Thymelaeaceae	*Synaptolepis oliveriana* Gilg	NE1	shrub, liana		CD, G, In, Mp, Na, Z	South Africa KN	
** Vahliaceae **	**Vahlia capensis (L.f.) Thunb. subsp. macrantha (Klotzsch) Bridson**	E	herb (a), herb (p)		Mc, S, Z	Possibly Madagascar	
Verbenaceae	Chascanum angolense Moldenke subsp. zambesiacum (R.Fern.) R.Fern.	NE2+3	shrub, herb (p)		In	Malawi	
** Verbenaceae **	**Chascanum schlechteri (Gürke) Moldenke var. torrei Moldenke**	E	herb (p)	Y	Mp		2a
Verbenaceae	*Lantana swynnertonii* Moldenke	NE1	shrub		Mc, Z	Zimbabwe	3a, 3b
Vitaceae	*Cissus aristolochiifolia* Planch.	NE1	herb (c)		Na, Z	Malawi	3b
Vitaceae	*Cissus bathyrhakodes* Werderm.	NE1	herb (p)		CD, Mc, Z	Tanzania	
Vitaceae	*Cyphostemma barbosae* Wild & R.B.Drumm.	NE1	herb (geo)		Mp	eSwatini, South Africa KN, MP	2b

## Supplementary materials

Supplementary material 1 Annotated checklist of the endemic and near-endemic vascular plant taxa of Mozambique Data type: species data Explanation note: Explanatory notes in addition to those for Appendix 1: For “Sources”, F.Z. = Flora Zambesiaca; S.R.D.L. = Southern African Plant Red Data Lists (Izidine and Bandeira 2002); T.S.M. = Trees and Shrubs of Mozambique (Burrows et al. 2018). For “IUCN Status”, assessments in italics are awaiting publication; those marked with an asterisk (*) require updating. https://binary.pensoft.net/file/364107This dataset is made available under the Open Database License (http://opendatacommons.org/licenses/odbl/1.0/). The Open Database License (ODbL) is a license agreement intended to allow users to freely share, modify, and use this Dataset while maintaining this same freedom for others, provided that the original source and author(s) are credited.Iain Darbyshire, Jonathan Timberlake, Jo Osborne, Saba Rokni, Hermenegildo Matimele, Clayton Langa, Castigo Datizua, Camila de Sousa, Tereza Alves, Alice Massingue, Jeneen Hadj-Hammou, Sonia Dhanda, Toral Shah, Bart Wursten

Supplementary material 2 Taxa that were considered for their potential status as near-endemics for Mozambique in preparation of the checklist but that do not meet the criteria set out in the Materials and methods Data type: species data https://binary.pensoft.net/file/364117This dataset is made available under the Open Database License (http://opendatacommons.org/licenses/odbl/1.0/). The Open Database License (ODbL) is a license agreement intended to allow users to freely share, modify, and use this Dataset while maintaining this same freedom for others, provided that the original source and author(s) are credited.Iain Darbyshire, Jonathan Timberlake, Jo Osborne, Saba Rokni, Hermenegildo Matimele, Clayton Langa, Castigo Datizua, Camila de Sousa, Tereza Alves, Alice Massingue, Jeneen Hadj-Hammou, Sonia Dhanda, Toral Shah, Bart Wursten

## References

[B1] Abdelaal M Fois M Fenu G Bacchetta G (2018) Critical checklist of the endemic vascular plants of Egypt. Phytotaxa 360(1): 19–34. 10.11646/phytotaxa.360.1.2

[B2] African Plant Database (2019) African Plant Database (version 3.4.0). Conservatoire et Jardin botaniques de la Ville de Genève and South African National Biodiversity Institute, Pretoria. http://www.ville-ge.ch/musinfo/bd/cjb/africa/ [accessed 26.06.2019]

[B3] Angiosperm Phylogeny Group (2016) An update of the Angiosperm Phylogeny Group classification for the orders and families of flowering plants: APG IV. Botanical Journal of the Linnean Society 181(1): 1–20. 10.1111/boj.12385

[B4] Bachman S Moat J Hill AW de la Torre J Scott B (2011) Supporting red list threat assessments with GeoCAT: Geospatial conservation assessment tool. ZooKeys 150: 117–126. 10.3897/zookeys.150.2109 PMC3234434 22207809

[B5] Bandeira S Bolnick D Barbosa F (1997) Flores nativas do Sul de Moçambique / Wild flowers of Southern Mozambique. Universidade Eduardo Mondlane, Maputo, 1–258.

[B6] Bayliss J Monteiro J Fishpool L Congdon C Bampton I Bruessow C Matimele H Banze A Timberlake J (2010) Biodiversity and Conservation of Mount Inago, Mozambique. Report for Darwin Initiative Award 15/036: Monitoring and Managing Biodiversity Loss in South-East Africa’s Montane Ecosystems, 1–32.

[B7] Bayliss J Timberlake J Branch W Bruessow C Collins S Congdon C Curran M de Sousa C Dowsett R Dowsett-Lemaire F Fishpool L Harris T Herrmann E Georgiadis S Kopp M Liggitt B Monadjem A Patel H Ribeiro D Spottiswoode C Taylor P Willcock S Smith P (2014) The discovery, biodiversity and conservation of Mabu forest – The largest medium-altitude rainforest in southern Africa. Oryx 48(2): 177–185. 10.1017/S0030605313000720

[B8] Beentje H (2012) Flora of Tropical East Africa completed. Kew Scientist 42: 1.

[B9] Beentje H (2016) Tropical African floras: Progress, gaps, and future. Symbolae Botanicae Upsalienses 38: 101–119.

[B10] Bertolini A (1849) Miscellanea Botanica Octava. Novi Commentarii Academiae Scientiarum Instituti Bononiensis 9: 573–590.

[B11] Borokini TI (2014) A systematic compilation of endemic flora in Nigeria for conservation management. Journal of Threatened Taxa 6(11): 6406–6426. 10.11609/JoTT.o4010.6406-26

[B12] Brenan JPM (1970) Leguminosae (Mimosoideae). In: Brenan JPM (Ed.) Flora Zambesiaca 3(1).Crown Agents for Oversea Governments and Administrations, London, 1–153.

[B13] Bridson DM Verdcourt B (2003) Rubiaceae (part 3). In: Pope GV (Ed.) Flora Zambesiaca 5(3).Royal Botanic Gardens, Kew, 379–720.

[B14] Burgess N D’Amico Hales J Underwood E Dinerstein E Olson D Itoua I Schipper J Ricketts T Newman K (2004) Terrestrial Ecoregions of Africa and Madagascar: A Conservation Assessment. WWF/Island Press, Washington, USA.

[B15] Burrows J Timberlake J (2011) Mozambique’s centres of endemism, with special reference to the Rovuma Centre of Endemism of NE Mozambique and SE Tanzania. South African Journal of Botany 77: 518. 10.1016/j.sajb.2011.03.003

[B16] Burrows J Burrows S Lötter M Schmidt E (2018) Trees and Shrubs of Mozambique. Publishing Print Matters, Noordhoek, Cape Town, 1–1114.

[B17] Carter S Lavranos JJ Newton LE Walker CC (2011) Aloes: the definitive guide. Royal Botanic Gardens, Kew, 1–720.

[B18] Cheek M Chipanga H Darbyshire I (2018) Notes on the plant endemics of the quartzitic slopes of Mt Chimanimani (Mozambique and Zimbabwe), and a new, Critically Endangered species, *Empogona jenniferae* (Rubiaceae-Coffeeae). Blumea 63: 87–92. 10.3767/blumea.2018.63.01.08

[B19] Christenhusz MJM Reveal JL Farjon A Gardiner MF Mill RR Chase MW (2011) A new classification and linear sequence of extant gymnosperms. Phytotaxa 19(1): 55–70. 10.11646/phytotaxa.19.1.3

[B20] Clark VR Barker NP Mucina L (2011) The Great Escarpment of southern Africa: A new frontier for biodiversity exploration. Biodiversity and Conservation 20(12): 2543–2561. 10.1007/s10531-011-0103-3

[B21] Clark VR Timberlake JR Hyde MA Mapaura A Coates Palgrave M Wursten BT Ballings P Burrows JE Linder HP McGregor GK Chapano C Plowes DCH Childes SL Dondeyne S Müller T Barker NP (2017) A first comprehensive account of floristic diversity and endemism of the Nyanga Massif, Manica Highlands (Zimbabwe-Mozambique). Kirkia 19: 1–53. https://www.researchgate.net/publication/317258598_A_first_comprehensive_account_of_floristic_diversity_and_endemism_on_the_Nyanga_massif_Manica_highlands_Zimbabwe-Mozambique

[B22] Clarke GP (1998) A new regional centre of endemism in Africa. In: Huxley CR Lock JM Cutler DF (Eds) Chorology, taxonomy and ecology of the Floras of Africa and Madagascar.Royal Botanic Gardens, Kew, 53–65.

[B23] Clarke GP (2001) The Lindi local centre of endemism in SE Tanzania. Systematics and Geography of Plants 71(2): 1063–1072. 10.2307/3668738

[B24] Crisp MD Laffan S Linder HP Monro A (2001) Endemism in the Australian flora. Journal of Biogeography 28(2): 183–198. 10.1046/j.1365-2699.2001.00524.x

[B25] Da Silva MC Izidine S Amude AB (2004) A preliminary checklist of the vascular plants of Mozambique. Southern African Botanical Diversity Network Report No. 30. SABONET, Pretoria, 1–183.

[B26] Darbyshire I Vollesen K Ensermu Kelbessa (2015) Acanthaceae (Part 2). In: Timberlake JR Martins ES (Eds) Flora Zambesiaca 8(6).Royal Botanic Gardens, Kew, 1–314.

[B27] Darbyshire I Anderson S Asatryan A Byfield A Cheek M Clubbe C Ghrabi Z Harris T Heatubun CD Kalema J Magassouba S McCarthy B Milliken W de Montmollin B Nic Lughadha E Onana JM Saïdou D Sârbu A Shrestha K Radford EA (2017) Important Plant Areas: Revised selection criteria for a global approach to plant conservation. Biodiversity and Conservation 26(8): 1767–1800. 10.1007/s10531-017-1336-6

[B28] Darbyshire I Burrows JE Alves MT Chelene I Datizua C de Sousa C Fijamo V Langa C Massingue AO Massunde J Matimele HA Mucaleque PA Osborne J Rokni S Sitoe P (2019) *Grewia transzambesica* The IUCN Red List of Threatened Species 2019: e.T32170A136537949. 10.2305/IUCN.UK.2019-2.RLTS.T32170A136537949.en

[B29] Dauby G Zaiss R Blach-Overgaard A Catarino L Damen T Deblauwe V Dessein S Dransfield J Droissart V Duarte MC Engledow H Fadeur G Figueira R Gereau RE Hardy OJ Harris DJ de Heij J Janssens S Klomberg Y Ley AC Mackinder BA Meerts P van de Poel JL Sonké B Sosef MSM Stévart T Stoffelen P Svenning J-C Sepulchre P van der Burgt X Wieringa JJ Couvreur TLP (2016) RAINBIO: A mega-database of tropical African vascular plants distributions. PhytoKeys 74: 1–18. 10.3897/phytokeys.74.9723 PMC5234546 28127234

[B30] Dinerstein E Olson D Joshi A Vynne C Burgess ND Wikramanayake E Hahn N Palminteri S Hedao P Noss R Hansen M Locke H Ellis EC Jones B Barber CV Hayes R Kormos C Martin V Crist E Sechrest W Price L Baillie JEM Weeden D Suckling K Davis C Sizer N Moore R Thau D Birch T Potapov P Turubanova S Tyukavina A de Souza N Pintea L Brito JC Llewellyn OA Miller AG Patzelt A Ghazanfar SA Timberlake J Klöser H Shennan-Farpón Y Kindt R Barnekow Lillesø J-P van Breugel P Graudal L Voge M Al-Shammari KF Saleem M (2017) An Ecoregion-Based Approach to Protecting Half the Terrestrial Realm. Bioscience 67(6): 534–545. 10.1093/biosci/bix014 28608869 PMC5451287

[B31] Diniz MA (1988) Bignoniaceae. In: Launert E (Ed.) Flora Zambesiaca 8(3).Flora Zambesiaca Managing Committee, London, 61–85.

[B32] Dorr LJ Wurdack KJ (2018) A new disjunct species of *Eriolaena* (Malvaceae, Dombeyoideae) from Continental Africa. PhytoKeys 111: 11–16. 10.3897/phytokeys.111.29303 PMC6234222 30473615

[B33] Downes E Darbyshire I (2017) *Coleus namuliensis* and *Coleus caudatus* (Lamiaceae): A new species and a new combination in the Afromontane flora of Mozambique and Zimbabwe. Blumea 62(3): 168–173. 10.3767/blumea.2017.62.03.02

[B34] Exell AW Wild H (Eds) (1960) Flora Zambesiaca 1(1). Crown Agents for Oversea Governments and Administrations, London, 1–336.

[B35] Fernandes A (1978) Lythraceae. In: Launert E (Ed.) Flora Zambesiaca 4.Flora Zambesiaca Managing Committee, London, 276–323.

[B36] Frodin D (2001) Guide to Standard Floras of the World. Second Edition. Cambridge University Press, Cambridge, 1–1100. 10.1017/CBO9780511541803

[B37] GBIF.org (2019) GBIF Home Page. https://www.gbif.org [accessed 21.10.2019]

[B38] Grace OM Klopper RR Figueiredo E Smith GF (2011) The aloe names book. Strelitzia 28. South African National Biodiversity Institute, Pretoria; and the Royal Botanic Gardens, Kew, 1–232.

[B39] Harris T Darbyshire I Polhill R (2011) New species and range extensions from Mt Namuli, Mt Mabu and Mt Chiperone in northern Mozambique. Kew Bulletin 66(2): 241–251. 10.1007/s12225-011-9277-9

[B40] Hyde MA Wursten BT Ballings P Coates Palgrave M (2019a) Flora of Mozambique. https://www.mozambiqueflora.com/ [accessed 21.03.2019]

[B41] Hyde MA Wursten BT Ballings P Coates Palgrave M (2019b) Flora of Zimbabwe. https://www.zimbabweflora.co.zw [accessed 21.03.2019]

[B42] Işik K (2011) Rare and endemic species: Why are they prone to extinction? Turkish Journal of Botany 35: 411–417.

[B43] IUCN (2012) IUCN Red List Categories and Criteria. Version 3.1, 2^nd^ edn. IUCN Species Survival Commission, Gland. 1–32. http://www.iucnredlist.org/technical-documents/categories-and-criteria/2001-categories-criteria [accessed 26.06.2019]

[B44] IUCN (2016) A global standard for the identification of Key Biodiversity Areas, version 1.0, 1^st^ edn. IUCN, Gland. https://portals.iucn.org/library/node/46259 [accessed 31.05.2019]

[B45] IUCN (2019) The IUCN Red List of Threatened Species. Version 2019-2. https://newredlist.iucnredlist.org/ [accessed 12.08.2019]

[B46] IUCN SSC Southern African Plant Specialist Group (2017) IUCN SSC Southern African Plant Specialist Group: 2016–17 report. https://www.iucn.org/sites/dev/files/2016-2017_southern_african_plant_sg_report.pdf [Accessed 28.06.2019]

[B47] Izidine S Bandeira SO (2002) Mozambique. In: Golding JS (Ed.) Southern African Plant Red Data Lists.Southern African Botanical Diversity Network Report No. 14. SABONET, Pretoria, 43–60.

[B48] Joppa LN Butchart SH Hoffmann M Bachman SP Akçakaya HR Moat JF Böhm M Holland RA Newton A Polidoro B Hughes A (2016) Impact of alternative metrics on estimates of extent of occurrence for extinction risk assessment. Conservation Biology 30(2): 362–370. 10.1111/cobi.12591 26183938

[B49] Lewis G Schrire B Mackinder B Lock M (2005) Legumes of the World. Royal Botanic Gardens, Kew, 1–577.

[B50] Linder HP Lehmann CER Archibald S Osborne CP Richardson DM (2017) Global grass (Poaceae) success underpinned by traits facilitating colonization, persistence and habitat transformation. Biological Reviews of the Cambridge Philosophical Society 93(2): 1125–1144. 10.1111/brv.12388 29230921

[B51] Loffler L Loffler P (2005) Swaziland Tree Atlas – including selected shrubs and climbers. Southern African Botanical Diversity Network Report No. 35. SABONET, Pretoria, 1–199.

[B52] Mapaura A (2002) Endemic plant species of Zimbabwe. Kirkia 18: 117–148. https://www.jstor.org/stable/23502383 [accessed 26.06.2019]

[B53] Matimele H (2019) Mozambique Endemic and Near-Endemic Red Listed Plant Species. Version 1.6. Herbarium LMA: Agricultural Research Institute of Mozambique. Occurrence dataset: 10.15468/8enzjm [accessed 05.06.2019]

[B54] Matimele HA Raimondo D Bandeira S Burrows JE Darbyshire I Massingue AO Timberlake J (2016) *Emicocarpus fissifolius* The IUCN Red List of Threatened Species 2016: e.T85955108A85955412. 10.2305/IUCN.UK.2016-3.RLTS.T85955108A85955412.en

[B55] MITADER (2015) National strategy and action plan of biological diversity of Mozambique (2015–2035). Ministério da Terra, Ambiente e Desenvolvimento Rural (MITADER), Républica de Moçambique. https://www.cbd.int/doc/world/mz/mz-nbsap-v3-en.pdf [accessed 26.06.2019]

[B56] Müller T Mapaura A Wursten B Chapano C Ballings P Wild R (2012) Vegetation Survey of Mount Gorongosa. Occasional Publications in Biodiversity No. 23: 1–54. Biodiversity Foundation for Africa, Bulawayo, Zimbabwe. http://www.gorongosa.org/our-story/science/reports/vegetation-survey-mount-gorongosa [accessed 26.06.2019]

[B57] Olson DM Dinerstein E Wikramanayake ED Burgess ND Powell GVN Underwood EC D’Amico JA Itoua I Strand HE Morrison JC Loucks CJ Allnutt TF Ricketts TH Kura Y Lamoreux JF Wettengel WW Hedao P Kassem KR (2001) Terrestrial ecoregions of the world: A new map of life on Earth. Bioscience 51(11): 933–938. 10.1641/0006-3568(2001)051[0933:TEOTWA]2.0.CO;2

[B58] Onana JM (2013) Synopsis de especès végétales vasculaires endémiques et rares du Cameroun. Check-liste pour la gestation durable et la conservation de la biodiversité. In: Onana JM (Ed.) Flore du Cameroun, Vol.40. Ministère de la Recherche Scientifique et de l’Innovation (MINRESI), Yaoundé, 1–279.

[B59] Orsenigo S Montagnani C Fenu G Gargano D Peruzzi L Abeli T Alessandrini A Bacchetta G Bartolucci F Bovio M Brullo C Brullo S Carta A Castello M Cogoni D Conti F Domina G Foggi B Gennai M Gigante D Iberite M Lasen C Magrini S Perrino EV Prosser F Santangelo A Selvagii A Stinca A Vagge I Villani M Wagensommer RP Wilhalm T Tartaglini N Duprè E Blasi C Rossi G (2018) Red Listing plants under full national responsibility: Extinction risk and threats in the vascular flora endemic to Italy. Biological Conservation 224: 213–222. 10.1016/j.biocon.2018.05.030

[B60] Peters WCH (1861) Naturwissenschaftliche Reise nach Mossambique. VI: Botanik. G. Reimer, Berlin, 1–584.

[B61] POWO (2019) Plants of the World Online. Facilitated by the Royal Botanic Gardens, Kew. http://www.plantsoftheworldonline.org/ [accessed 21.10.2019]

[B62] Pteridophyte Phylogeny Group (PPG) (2016) A community-derived classification for extant lycophytes and ferns. Journal of Systematics and Evolution 54(6): 563–603. 10.1111/jse.12229

[B63] Reynolds GW (1950) The Aloes of South Africa. Trustees of the Aloes of South Africa Book Fund, Johannesburg, 1–520.

[B64] Ricketts TH Dinerstein E Boucher T Brooks TM Butchart SHM Hoffmann M Lamoreux JF Morrison J Parr M Pilgrim JD Rodrigues ASL Sechrest W Wallace GE Berlin K Bielby J Burgess ND Church DR Cox N Knox D Loucks C Luck GW Master LL Moore R Naidoo R Ridgely R Schatz GE Shire G Strand H Wettengel W Wikramanayake E (2005) Pinpointing and preventing imminent extinctions. Proceedings of the National Academy of Sciences of the United States of America 102(51): 18497–18501. 10.1073/pnas.0509060102 16344485 PMC1311739

[B65] Smith TJ (2005) Important Plant Areas in southern Africa. Combined proceedings of workshops held in Mozambique, Namibia and South Africa. Southern African Botanical Network Report No. 39. SABONET, Pretoria, 1–52.

[B66] Sosef M Dauby G Blach-Overgaard A van der Burgt X Catarino L Damen T Deblauwe V Dessein S Dransfield J Drioissart V Duarte MC Engledow H Fadeur G Figueira R Gereau RE Hardy OJ Harris DJ de Heij J Janssens S Klomberg Y Ley AC Mackinder BA Meerts P van de Poel JL Sonké B Stévart T Stoffelen P Svenning J-C Sepulchre P Zaiss R Wieringa JJ Couvreur TLP (2017) Exploring the floristic diversity of tropical Africa. BMC Biology 15(1): 15 10.1186/s12915-017-0356-8 28264718 PMC5339970

[B67] South African National Biodiversity Institute (2017) Red List of South African Plants, Version 2017.1. South African National Biodiversity Institute (SANBI). http://redlist.sanbi.org [accessed 21.03.2019]

[B68] South African National Biodiversity Institute (2019) Botanical Database of Southern Africa (BODATSA). http://posa.sanbi.org/ [accessed 21.10.2019]

[B69] Stevens PF (2001 onwards) Angiosperm Phylogeny Website. Version 14, July 2017 [and more or less continuously updated since]. http://www.mobot.org/MOBOT/research/APweb/ [accessed 21.03.2019]

[B70] Stone RD (2014) The species-rich paleotropical genus *Memecylon* (Melastomataceae): Molecular phylogenetics and revised infrageneric classification of the African species. Taxon 63(3): 539–561. 10.12705/633.10

[B71] Stone RD Mona IG Ramdhani S (2018) Revised treatment of Mozambican *Memecylon* (Melastomataceae–Olisbeoideae), with descriptions of four new species in M. section Buxifolia Phytotaxa 331(2): 151–168. 10.11646/phytotaxa.331.2.1

[B72] Strugnell AM (2002) Endemics of Mt Mulanje. The endemic spermatophytes of Mt Mulanje, Malawi. Systematics and Geography of Plants 72: 11–26. https://www.jstor.org/stable/pdf/3668760.pdf

[B73] Strugnell AM (2006) A checklist of the Spermatophytes of Mount Mulanje, Malawi. Scripta Botanica Belgica 34. National Botanic Garden of Belgium, Meise, 1–199.

[B74] Thiers B [continuously updated] Index Herbariorum: A global directory of public herbaria and associated staff. New York Botanical Garden’s Virtual Herbarium. http://sweetgum.nybg.org/science/ih/ [accessed 26.06.2019]

[B75] Timberlake JR Martins ES (2015) Flora Zambesiaca 8(6). Royal Botanic Gardens, Kew, 1–314.

[B76] Timberlake J Golding JS Smith P (2006) A preliminary analysis of endemic and threatened plants of the Flora Zambesiaca area. In: Ghazanfar SA Beentje H (Eds) Taxonomy and Ecology of African Plants and their Conservation and Sustainable Use.Proceedings of the 17^th^ AETFAT Congress, Addis Ababa, Ethiopia. Royal Botanic Gardens, Kew, 749–760.

[B77] Timberlake J Bayliss J Alves T Baena S Francisco J Harris T de Sousa C (2007) The biodiversity and conservation of Mount Chiperone, Mozambique. Report produced under the Darwin Initiative award 15/036. Royal Botanic Gardens, Kew, 1–33. http://www.biofund.org.mz/wp-content/uploads/2018/12/1544778472-F2339.Darwin%20Initiative%20Award%2015%20036%20Monitoring%20and%20Managing%20Biodiversity%20Loss%20in%20Sout_2007_Timberlake_Et_Al_Chiperone.Pdf

[B78] Timberlake J Dowsett-Lemaire F Bayliss J Alves T Baena S Bento C Cook K Francisco J Harris T Smith P de Sousa C (2009) Mt Namuli, Mozambique: Biodiversity and Conservation. Report produced under the Darwin Initiative award 15/036. Royal Botanic Gardens, Kew, United Kingdom, 1–115. http://www.biofund.org.mz/biblioteca_virtual/mt-namuli-mozambique-biodiversity-and-conservation/

[B79] Timberlake J Goyder D Crawford F Pascal O (2010) Coastal Dry Forests in Cabo Delgado Province, Northern Mozambique: Botany and Vegetation. Report for ProNatura International. Royal Botanic Gardens, Kew, 1–92.

[B80] Timberlake J Goyder D Crawford F Burrows J Clarke GP Luke Q Matimele H Müller T Pascal O de Sousa C Alves T (2011) Coastal dry forests in northern Mozambique. Plant Ecology and Evolution 144(2): 126–137. 10.5091/plecevo.2011.539

[B81] Timberlake J Bayliss J Dowsett-Lemaire F Congdon C Branch B Collins S Curran M Dowsett RJ Fishpool L Francisco J Harris T Kopp M de Sousa C (2012) Mt Mabu, Mozambique: biodiversity and conservation. Report for Darwin Initiative Award 15/036: Monitoring and Managing Biodiversity Loss in South-East Africa’s Montane Ecosystems. Royal Botanic Gardens, Kew, 1–94. https://www.kew.org/sites/default/files/Mabu%20report_Final%202012_0.pdf

[B82] Timberlake J Darbyshire I Cheek M Banze A Fijamo V Massunde J Chipanga H Muassinar D (2016a) Plant Conservation in Communities on the Chimanimani footslopes. Report produced under Darwin Initiative Award 2380: Balancing Conservation and Livelihoods in the Chimanimani Forest Belt, Mozambique. Royal Botanic Gardens, Kew, 1–69. https://www.kew.org/sites/default/files/Chimanimani%20Darwin%20report%2C%20FINAL.pdf

[B83] Timberlake JR Darbyshire I Wursten B Hadj-Hammou J Ballings P Mapaura A Matimele H Banze A Chipanga H Muassinar D Massunde M Chelene I Osborne J Shah T (2016b) Chimanimani Mountains: Botany and Conservation. Report produced under CEPF Grant 63512. Royal Botanic Gardens, Kew, 1–95. https://www.kew.org/sites/default/files/Chimanimani%20CEPF%20report%202016_FINAL.pdf

[B84] Van Wyk AE (1996) Biodiversity of the Maputaland Centre. In: van der Maesen LJG, van der Burgt XM, van Medenbach de Rooy JM (Eds) The Biodiversity of African Plants; Proceedings XIVth AETFAT Congress 22–27 August 1994, Wageningen, The Netherlands, 198–207. 10.1007/978-94-009-0285-5_26

[B85] Van Wyk AE Smith GF (2001) Regions of Floristic Endemism in Southern Africa. A review with emphasis on succulents. Umdaus Press, Hatfield, South Africa, 1–199.

[B86] Verdcourt B (1994) Lythraceae. In: Polhill RM (Ed.) Flora of Tropical East Africa.A.A. Balkema, Rotterdam, 1–62.

[B87] White F (1983) Vegetation of Africa. A Descriptive Memoir to Accompany the UNESCO/AETFAT/UNSO Vegetation Map of Africa. Natural Resources Research 20. UNESCO, Paris, 1–356.

[B88] Wild H (1964) The endemic species of the Chimanimani Mountains and their significance. Kirkia 4: 125–157. https://www.jstor.org/stable/23501005

[B89] Wursten B Timberlake J Darbyshire I (2017) The Chimanimani Mountains: an updated checklist. Kirkia 19: 70–100. http://www.biofund.org.mz/biblioteca_virtual/the-chimanimani-mountains-un-updated-checklist/

